# *Ruminococcus torques* is a keystone degrader of intestinal mucin glycoprotein, releasing oligosaccharides used by *Bacteroides thetaiotaomicron*

**DOI:** 10.1128/mbio.00039-24

**Published:** 2024-07-08

**Authors:** Sadie R. Schaus, Gabriel Vasconcelos Pereira, Ana S. Luis, Emily Madlambayan, Nicolas Terrapon, Matthew P. Ostrowski, Chunsheng Jin, Bernard Henrissat, Gunnar C. Hansson, Eric C. Martens

**Affiliations:** 1 Department of Microbiology and Immunology, University of Michigan, Ann Arbor, Michigan, USA; 2 Department of Medical Biochemistry and Cell Biology, University of Gothenburg, Gothenburg, Sweden; 3 Centre National de la Recherche Scientifique, Aix-Marseille University, Marseille, France; 4 Institut National de Recherche pour l’Agriculture, l’Alimentation et l’Environnement, Marseille, France; 5 Proteomics Core Facility at Sahlgrenska Academy, University of Gothenburg, Gothenburg, Sweden; 6 Department of Biotechnology and Biomedicine, Technical University of Denmark, Lyngby, Denmark; University of Hawaii at Manoa, Honolulu, Hawaii, USA

**Keywords:** microbiota, inflammatory bowel disease, mucus, glycoprotein, *Ruminococcus torques*

## Abstract

Symbiotic interactions between humans and our communities of resident gut microbes (microbiota) play many roles in health and disease. Some gut bacteria utilize mucus as a nutrient source and can under certain conditions damage the protective barrier it forms, increasing disease susceptibility. We investigated how *Ruminococcus torques—*a known mucin degrader that has been implicated in inflammatory bowel diseases (IBDs)—degrades mucin glycoproteins or their component *O*-linked glycans to understand its effects on the availability of mucin-derived nutrients for other bacteria. We found that *R. torques* utilizes both mucin glycoproteins and released oligosaccharides from gastric and colonic mucins, degrading these substrates with a panoply of mostly constitutively expressed, secreted enzymes. Investigation of mucin oligosaccharide degradation by *R. torques* revealed strong α-L-fucosidase, sialidase and β1,4-galactosidase activities. There was a lack of detectable sulfatase and weak β1,3-galactosidase degradation, resulting in accumulation of glycans containing these structures on mucin polypeptides. While the Gram-negative symbiont, *Bacteroides thetaiotaomicron* grows poorly on mucin glycoproteins, we demonstrate a clear ability of *R. torques* to liberate products from mucins, making them accessible to *B. thetaiotaomicron*. This work underscores the diversity of mucin-degrading mechanisms in different bacterial species and the probability that some species are contingent on others for the ability to more fully access mucin-derived nutrients. The ability of *R. torques* to directly degrade a variety of mucin and mucin glycan structures and unlock released glycans for other species suggests that it is a keystone mucin degrader, which might contribute to its association with IBD.

**IMPORTANCE:**

An important facet of maintaining healthy symbiosis between host and intestinal microbes is the mucus layer, the first defense protecting the epithelium from lumenal bacteria. Some gut bacteria degrade the various components of intestinal mucins, but detailed mechanisms used by different species are still emerging. It is imperative to understand these mechanisms as they likely dictate interspecies interactions and may illuminate species associated with bacterial mucus damage and subsequent disease susceptibility. *Ruminococcus torques* is positively associated with IBD in multiple studies. We identified mucin glycan-degrading enzymes in *R. torques* and found that it shares mucin degradation products with another species of gut bacteria, *Bacteroides thetaiotaomicron*. Our findings underscore the importance of understanding mucin degradation mechanisms in different gut bacteria and their consequences on interspecies interactions, which may identify keystone bacteria that disproportionately affect mucus damage and could therefore be key players in effects that result from reductions in mucus integrity.

## INTRODUCTION

Mammals have evolved a series of physical and immunological defenses to promote appropriate separation between the gut microbiota and intestinal tissue. Some bacteria—especially pathogens—can disrupt these defenses and promote disease (1). Secreted mucus is a critical component of the host defense in the colon and forms a physical barrier protecting epithelial cells. Previous studies have demonstrated the ability of some gut bacteria to affect mucus properties *in vivo*, particularly in the context of a fiber-deficient diet, leading to decreased mucus thickness observed in fixed tissues or increased mucus penetrability and decreased growth rate of the inner mucus layer in tissue explants (2–5). Some mucin degraders are prevalent in the human population and have known beneficial effects, including *Akkermansia muciniphila*, which notably supports metabolic health (6–8), and *Bacteroides thetaiotaomicron*, which degrades a multitude of dietary fibers and produces short-chain fatty acids (5, 9–11). However, increased abundance, activity, or presence of certain mucin-degrading bacteria has been associated with detrimental effects in some models, including increasing pathogen susceptibility (5, 12), development of spontaneous colitis like that which occurs in inflammatory bowel diseases (IBDs) (13), increased allergen sensitivity (14), and higher mortality due to carbapenem-associated graft vs. host disease (15).

Mammalian colonic mucus is predominantly composed of high-molecular weight (~2.5 MDa) mucin 2 (MUC2) monomers that are disulfide cross-linked to form a polymeric glycoprotein network (16). MUC2 harbors hundreds of structurally unique *O*-linked glycan structures (herein, *O*-glycans) attached to the MUC2 polypeptide at serine and threonine sequences, accounting for up to 80% of mucin total mass (16–18). Though composed of only five monosaccharides, the structural diversity and sterically hindered nature of these glycans attached to MUC2 create a complex and recalcitrant substrate requiring a large repertoire of enzymes to degrade (18). Adding to its complexity, *O*-glycan composition varies both between different segments of the gut and between individuals. Many *O-*glycans are decorated with capping residues, including fucose, sialic acid, and sulfate. In humans, fucosylated glycans decrease in abundance from the proximal to the distal gastrointestinal tract, while sialylated and sulfated glycans exhibit an opposite trend (19). As these residues are typically present at the non-reducing end, bacteria utilizing *O-*glycans as a nutrient source must be able to remove these capping residues or encode enzymes which tolerate their presence, in order to degrade the underlying *O-*glycan structure. Individual blood group status (A, B, AB, and H) is also reflected in *O*-glycans as terminal, non-reducing end sugar linkages (1).

Historically, readily available porcine gastric mucin (PGM) and PGM-derived substrates have been used to study mucin-degrading abilities of gut bacteria. Species known to degrade PGM to varying degrees include several species of *Bacteroides* (*Bacteroides fragilis*, *Bacteroides vulgatus*, *Bacteroides thetaiotaomicron*, and *Bacteroides caccae*), *Akkermansia muciniphila*, *Ruminococcus gnavus*, *Ruminococcus torques*, *Peptostreptococcus russellii*, and multiple *Clostridiales* and *Bifidobacterium* species (20–23). However, some mucin degraders can only access certain mucin components. For example, total growth yield of *B. thetaiotaomicron* on PGM glycoprotein is poor (24, 25) but is greatly increased on *O-*glycans chemically released from PGM (26). Many bacterial carbohydrate active enzymes (CAZymes) active on mucins have been identified (20, 27), although a comprehensive list of bacteria and enzymes involved in mucin degradation is still needed. Few commensal bacterial proteases targeting mucin glycoproteins have been identified but include M60-like metalloproteases (28, 29). Studies investigating growth on diverse mucin substrates, especially highly sulfated mucins from the mammalian colon, remain limited (30–33) but may reveal different and more physiologically relevant mechanisms due to their structural differences from PGM.

Notably, a previous study found that *R. torques*, *A. muciniphila*, *Bifidobacterium bifidum,* and *R. gnavus* all degrade human colonic MUC2, and *R. torques* was the most efficient of the species investigated (31). *R. torque*s is more prevalent and abundant in people with IBDs (31) and is one of only two bacterial species positively correlated with anti-*Saccharomyces cerevisiae* antibodies, a marker of Crohn’s disease (34). A recent prospective study following first-degree relatives of individuals with Crohn’s disease before diagnosis found that increased colonization with *R. torques* was the top microbial risk associated with developing Crohn’s disease (35). Previous work demonstrated that *R. torques* ferments PGM and degrades the blood group A and H antigen components of *O*-glycans and Lewis x and y antigens present in intestinal glycosphingolipids (36–38). Given the implication of *R. torques* as a mucin degrader associated with IBD (31, 35, 39), we aimed to characterize the mechanisms by which *R. torques* degrades both gastric and colonic mucin and the influence of this degradation on interspecies interactions.

## RESULTS

### *R. torques* grows on mucin substrates and isolated supernatants degrade mucin glycoproteins

**Fig 1:**
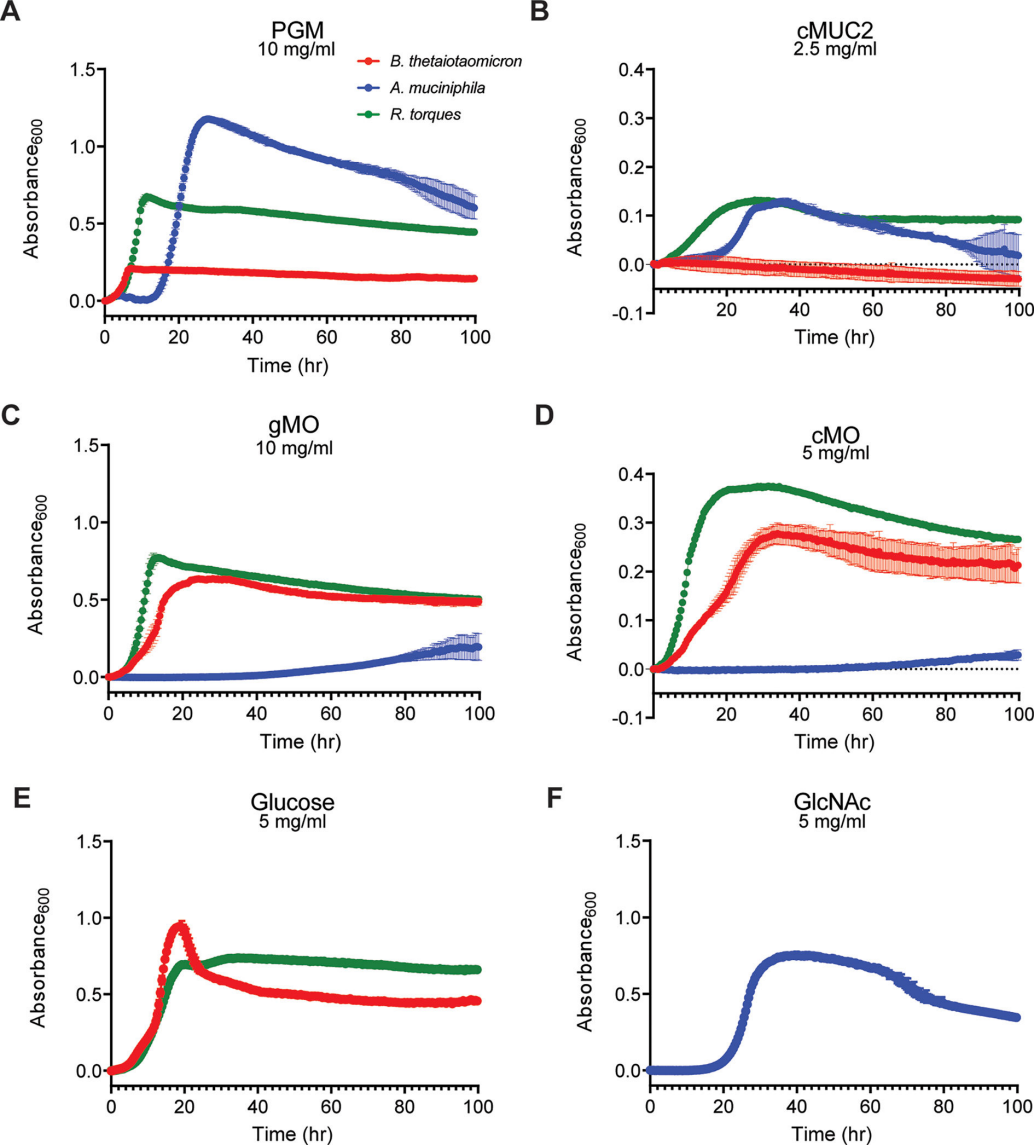
Variation in bacterial growth preferences for different mucin substrates. (A–F) Growth of three commensal gut bacterial species (*Bacteroides thetaiotaomicron*, *Akkermansia muciniphila*, and *R. torques*) measured anaerobically at 37°C by absorbance at 600 nm on porcine gastric mucin (PGM) (A; 10 mg/mL final, *n* = 3), mucin glycoprotein purified from porcine colonic mucosa (B; cMUC2, 2.5 mg/mL final, *n* = 3), released *O*-glycans from PGM (C; gMO, 10 mg/mL final, *n* = 3), and released *O*-glycans from cMUC2 (D; cMO, 5 mg/mL final, *n* = 2). Growths on glucose (E; 5 mg/mL final, *B. thetaiotaomicron* and *R. torques*, *n* = 3) and *N*-acetylglucosamine (F; GlcNAc, 5 mg/mL final, *A. muciniphila*, *n* = 3) were included as positive controls. Each point represents the average of the replicates, and error bars represent standard deviations. *B. thetaiotaomicron* was grown in Bacteroides minimal medium; *A. muciniphila* was grown in chopped meat medium; and *R. torques* was grown in yeast casitone fatty acid (YCFA) medium (see Materials and Methods for medium formula and references).

*R. torques* was previously found to degrade PGM and human MUC2 using biochemical approaches (31, 37). However, direct growth measurements of *R. torques* on various mucin and non-mucin substrates have not been reported. We assessed the ability of three species of gut bacteria, including a human *R. torques* isolate [*R. torques* VIII-239 (37)] to degrade mucin glycoproteins from pig stomach (PGM) or colonic MUC2 (cMUC2) and their chemically released *O-*glycans [gastric mucin *O*-glycans (gMO) or colonic mucin *O*-glycans (cMO)]. *Bacteroides thetaiotaomicron* and *Akkermansia muciniphila* are also known to degrade components of gastric mucin (24, 26, 40). Interestingly, these species displayed partially opposing preferences for gastric and colonic mucins or their component *O*-glycans, with *R. torques* displaying the broadest mucin-degradation abilities. On the mucin glycoprotein substrates, *A. muciniphila* and *R. torques* grew to a higher maximum absorbance at 600 nm (*A*_600_) than *B. thetaiotaomicron* (Fig. 1A and B). In contrast, *R. torques* and *B. thetaiotaomicron* grew to a higher max absorbance on the mucin *O*-glycans substrates than *A. muciniphila* (Fig. 1C and D). All strains grew on positive control substrates selected based on previous growth profiles (5) (Fig. 1E and F).

Growth experiments using 46 other host- and plant-derived polysaccharides and their constituent monosaccharides (41) suggest that *R. torques* is a mucin specialist. Strong growth (≥0.7 average net *A*_600_ increase) was only observed on glucose, fructose, and three mucin monosaccharides: galactose, *N*-acetylgalactosamine, and *N*-acetylglucosamine (Fig. S1). *R. torques* grew modestly on glucosamine and keratan sulfate (~0.3 and ~0.15 average net *A*_600_ increases, respectively) but did not demonstrate growth (≥0.1 net *A*_600_) on other substrates, with the exception of variable growth on laminarin and pectic galactan from lupin (Fig. S1). Because of its broad ability to degrade mucins and *O*-glycans from different regions of the gastrointestinal tract, we sought to understand the enzyme repertoire *R. torques* uses to degrade these substrates.

**Fig 2:**
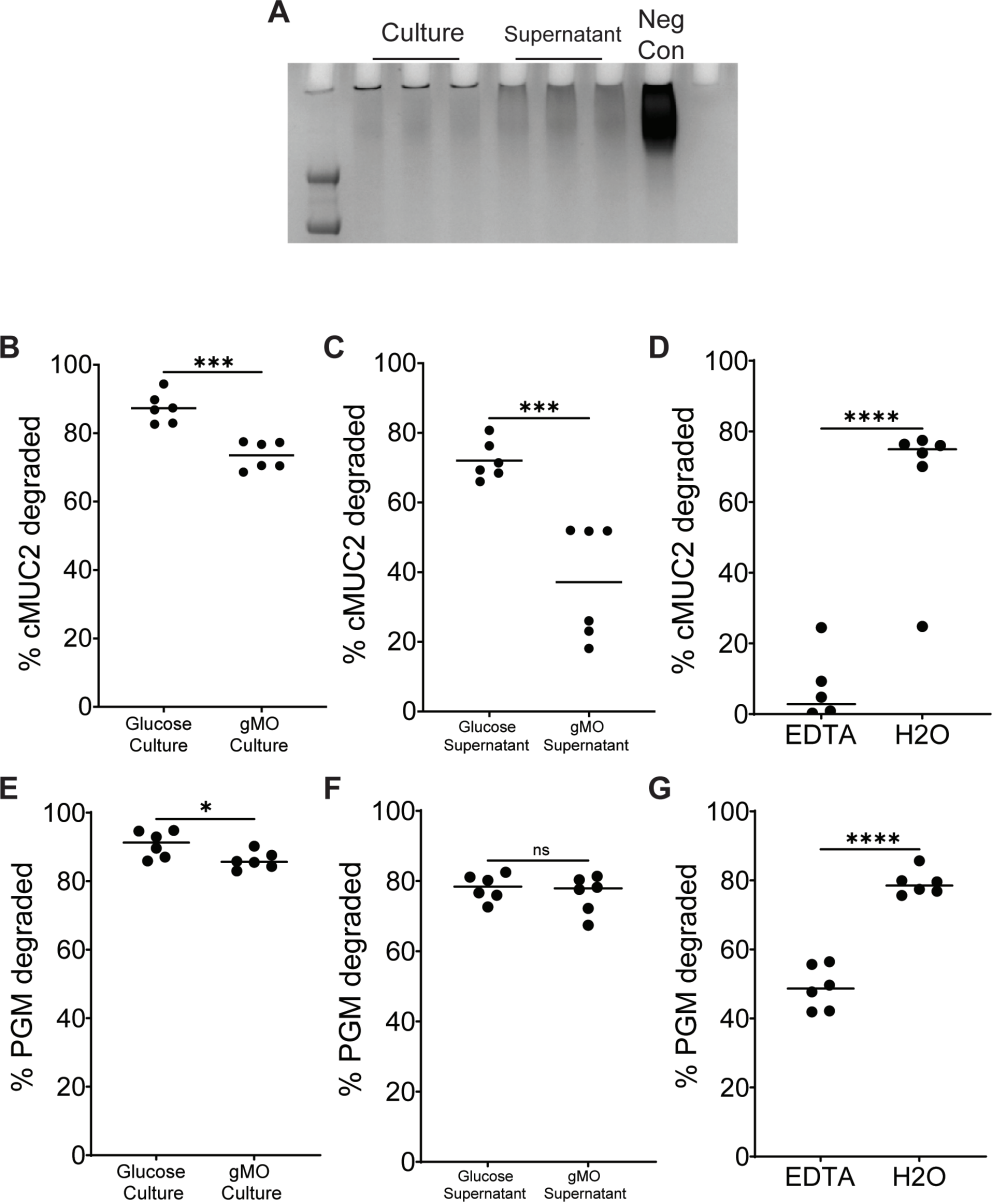
*R. torques* cultures and cell-free supernatants degrade gastric and colonic mucin glycoprotein. (A) Representative periodic acid-Schiff stained gel of samples of *R. torques* culture or supernatant degradation of cMUC2 after growth on glucose. Neg Con contains cMUC2 and yeast casitone fatty acid medium. (B and C) Percent cMUC2 degraded after *R*. *torques* growth in glucose or gastric mucin *O*-glycans (gMOs). Culture samples (B) or cell-free supernatants (C) from these cultures were exposed to cMUC2 for 48 h. cMUC2 degradation was quantified by visualizing the *R*. *torques*-digested cMUC2 on a 4%–12% Bis-Tris gel, staining with periodic acid-Schiff stain, and using densitometry to quantify the resulting bands using ImageJ. (D) Percent cMUC2 degraded by *R*. *torques* supernatants in the presence or absence of 10 mM EDTA. (E and F) Percent gastric mucin glycoprotein (PGM) degraded by *R*. *torques* culture (E) or cell-free supernatant (F) after previous growth on glucose or gMO. (G) Percent PGM degraded by *R*. *torques* supernatants in the presence or absence of 10 mM EDTA. Statistics were analyzed with unpaired, two-tailed *t*-tests. *P < 0.05, ***P < 0.001, ****P < 0.0001. ns, not significant.

We evaluated the ability of whole culture, containing cells and associated supernatant, or cell-free supernatant, to degrade cMUC2 or PGM using a PAGE gel assay to visualize high-molecular weight MUC2 domains with periodic acid-Schiff staining (Fig. 2A). *B. thetaiotaomicron* and other mucin-degrading *Bacteroides* exhibit substrate-specific activation of genes involved in utilizing *O*-glycans and other polysaccharides (5, 26, 42). Thus, we sought to understand whether *R. torques*’ prior growth substrates influence its cMUC2 degradation. Both whole culture and supernatant from *R. torques* grown on either glucose or gMO degraded cMUC2 (Fig. 2A through C; Fig. S2A and B). Notably, the glucose-grown supernatant samples degraded 72.0% of cMUC2 (Fig. 2C), suggesting *R. torques* constitutively produces and secretes sufficient mucin-degrading enzymes to reduce the molecular weight or glycosylation of the mucins tested, even when they were previously grown in the absence of mucins. Surprisingly, prior growth on gMO significantly decreased cMUC2 degradation compared to prior growth on glucose in both culture and supernatant samples (Fig. 2B and C). This suggests that growth on gMO suppresses the production or activity of critical mucin-degrading factors. Degradation of cMUC2 was also inhibited by EDTA, suggesting that key mucin-degrading enzymes are metal dependent (Fig. 2D; Fig. S2C).

PGM was degraded similarly to cMUC2 by culture and supernatant samples. However, prior growth on gMO did not inhibit supernatant degradation of PGM (Fig. 2E and F; Fig. S2D). EDTA inhibited PGM degradation although less than with cMUC2 (48.9% PGM degraded vs. 6.4% cMUC2 degraded) (Fig. 2G; Fig. S2E). The variable effects of EDTA inhibition or prior gMO growth on cMUC2 and PGM degradation suggest that some shared enzymes target both substrates, but additional enzymes more critical for cMUC2 degradation are more sensitive to these factors.

### Putative CAZymes in *R. torques* supernatant degrade mucin *O*-glycan linkages

**Fig 3:**
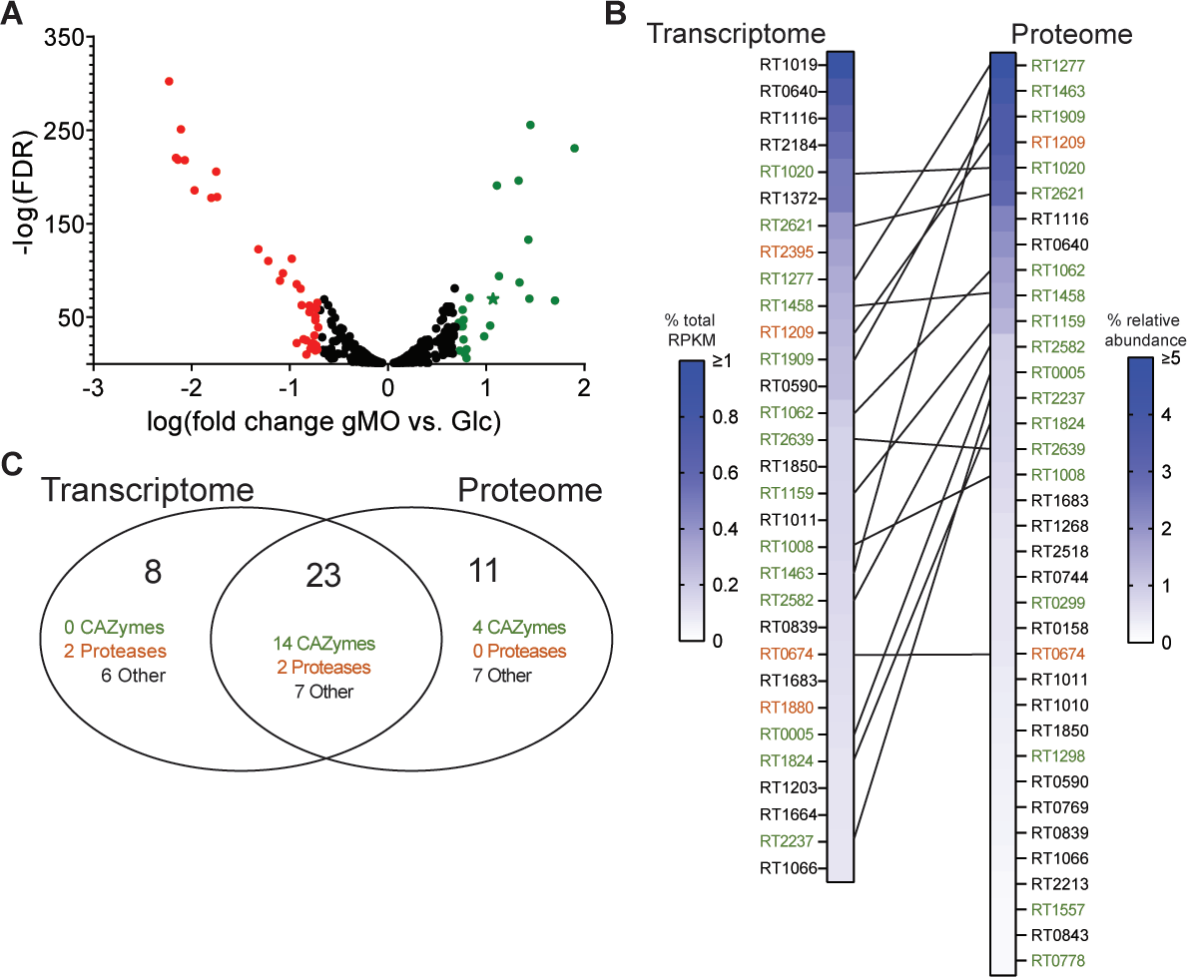
Candidate secreted mucin-degrading enzymes identified in the transcriptome and proteome of *R. torques*. (A) Genes significantly differentially expressed (*P* < 0.05) using RNA-sequencing after growth of *R. torques* on glucose or gMO. Points in green indicate genes upregulated (>5-fold) on gMO vs. glucose; points in red indicate genes downregulated (>5-fold) on gMO vs. glucose. Green star indicates predicted β-galactosidase. FDR, false discovery rate. (B) Top highly abundant genes or proteins from the *R. torques* transcriptome [RNA-seq, genes with predicted signal peptide and >0.1% of total reads per kilobase millions (RPKM)] or proteome (liquid chromatography-tandem mass spectrometry, proteins with predicted signal peptide and >0.1% average relative abundance). Lines connecting hits from the transcriptome and proteome indicate genes/proteins present as top hits in both data sets. Green locus tags represent predicted CAZymes or carbohydrate-binding module-containing proteins; orange locus tags represent predicted proteases. Transcriptome data, *n* = 3; proteome data, *n* = 6. (C) A Venn diagram showing combined top hits from transcriptome and proteome data sets.

To explore the observed inhibition of cMUC2 degradation after growth on gMO, we measured gene expression changes in *R. torques* grown on glucose or gMO by RNA-Seq. Only 25 genes were upregulated and 35 genes were downregulated during growth in gMO relative to the glucose reference (≥5-fold change, *P* < 0.05) (Fig. 3A; Table S1). A single putative mucin-degrading CAZyme (rumtor8_01403, predicted GH2 β-galactosidase) was ~12-fold upregulated during growth in gMO. However, this putative β-galactosidase does not harbor a predicted signal peptide.The list of differentially regulated functions includes genes related to bacteriophage, sugar or amino acid transporters, and metabolism of ATP, amine/amino acids, nucleotides, and sugars (Table S1), some of which may be involved in mucin product import.

To complement the transcriptomics, we precipitated proteins from *R. torques* supernatants after growth on glucose and analyzed them by liquid chromatography-tandem mass spectrometry (LC-MS/MS). Due to an observation that filtered (0.22 µM) *R. torques* supernatants displayed variable defects in degradation of cMUC2 (Fig. S3), we hypothesized that filtration may remove critical enzymes necessary for cMUC2 degradation. Comparing unfiltered, centrifuged samples to filtered samples, we first analyzed proteins containing a predicted signal peptide due to the observed mucin-degrading activity of *R. torques* supernatants (Fig. 2C and F) and to eliminate contaminants derived from lysed cells. We compiled a list of highly abundant proteins (>0.1% relative abundance) in at least two of the sample sets (Table S2). Interestingly, there was close similarity between the filtered samples and the unfiltered samples, suggesting that sample filtration did not remove a key enzyme but may disrupt protein structure or complexes. Thus, we compiled a list of proteins with a predicted signal peptide representing >0.1% relative abundance in at least two of three sample groups, yielding 34 putative mucin-targeting proteins, including 18 CAZymes and 2 proteases (Fig. 3B; Table S2).

To corroborate the proteomics results, we used our transcriptomic data to identify highly transcribed genes during *R. torques* growth on glucose. The top hits predicted to be secreted and representing >0.1% of all transcribed genes based on percentage of total RPKM included 31 genes: 14 CAZymes and 4 proteases (Fig. 3B). Importantly, 14 CAZymes and two proteases were shared with the proteomic data set (Fig. 3C; Table S3). These enzymes and the four highly expressed CAZymes unique to the proteomics data set represent top candidates for secreted *R. torques* mucin-degrading enzymes.

While our results suggest a critical role for secreted enzymes in *R. torques* mucin degradation, we assessed all predicted CAZymes in the *R. torques* genome regardless of presence of a signal peptide. We identified two putative CAZymes lacking a predicted signal peptide but present >0.1% relative abundance in the proteomics data set and/or >0.1% of total RPKM during growth on glucose from the transcriptomics data set: a carbohydrate esterase family 9 (CE9, rumtor8_00521) and a glycoside hydrolase family 112 (GH112, rumtor8_01112). The CE9 was detected >0.1% of the total relative abundance in all three proteomics samples and was just below the >0.1% of total RPKM threshold (0.096%). The GH112 was above this threshold in two of the three proteomics sample groups and represented 0.11% of the total RPKM. CE9 enzymes deacetylate *N*-acetylglucosamine-6-phosphate, and GH112 enzymes are galacto-*N*-biose and lacto-*N*-biose phosphorylases (43). These enzymes may be involved in *R. torques* mucin metabolism, and thus may contribute to increased cMUC2 degradation in whole culture samples compared to supernatant samples (Fig. 2A and B).

The 18 most highly expressed CAZymes from the transcriptomic and proteomics data belong to 12 different CAZy families (Fig. S4). Previously characterized enzymes from each of these families reflect expected catalytic roles in degrading mucin *O*-glycans: α*-N*-acetylglucosaminidase (GH89), β*-N*-acetylglucosaminidase (GH20, GH73, GH84, GH123), α-*N*-acetylgalactosaminidases (GH101 and GH31_18, GH36_3), sialidase (GH33), β-galactosidases (GH2), and α-L-fucosidases (GH29 and GH95) (43). Notably, 11 of 18 of these enzymes are predicted to contain at least one carbohydrate-binding module of family 32, which may play a critical role in mucin *O-*glycan binding (33). Many of these enzymes are also large, with 17 of 18 being >125 kDa, and 8 of 18 being >200 kDa (Table S3). Despite *R. torques* growth on and degradation of sulfated cMUC2 and cMO, there was a surprising lack of predicted sulfatase enzymes. Indeed, bioinformatic homology searches against the SulfAtlas HMM library failed to identify candidate sulfatases in the *R. torques* genome.

**Fig 4:**
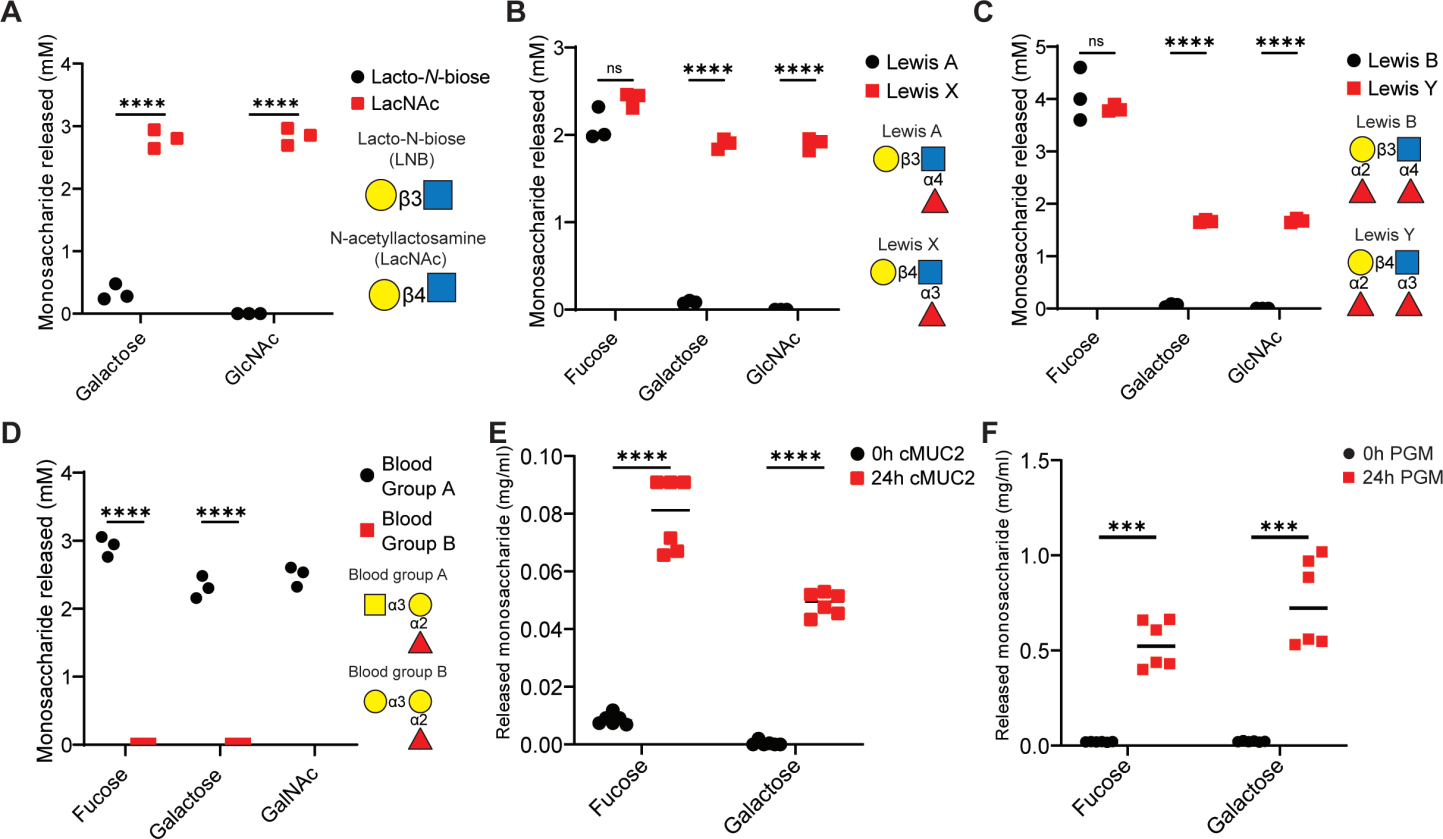
*R. torques* supernatant enzymes exhibit linkage specificity on model mucin glycans and release monosaccharides from intact mucin glycoprotein substrates. (A–D) Quantification of monosaccharides released from lacto-*N*-biose (LNB) or *N*-acetyllactosamine (LacNAc) (5 mM concentration each, (A), Lewis a or Lewis x antigens (2 mM final concentration each, (B), Lewis b or Lewis y antigens (2 mM final concentration each, (C), and blood group A or blood group B (2 mM final concentration each, (D) by ammonium sulfate precipitated proteins from *R. torques* supernatants after growth on glucose, measured by high-performance anion exchange chromatography with pulsed amperometric detection. For LNB samples, the galactose concentration found in the negative control was subtracted from concentrations calculated for supernatant reactions due to the presence of a species with a similar retention time in the no-enzyme control reaction. For A–D, statistical analyses were performed with unpaired, two-tailed *t*-tests comparing release of each monosaccharide between substrates in each panel. ***P < 0.001, ****P < 0.0001. (E and F) Concentration of fucose or galactose released after 24-h incubation of *R. torques* supernatants with cMUC2 (2.5 mg/mL final; (E) or PGM (10 mg/mL final; (F). For E–F, statistics were analyzed with paired, two-tailed *t*-tests comparing release of each monosaccharide between substrates in each panel. ***P < 0.001, ****P < 0.0001. ns, not significant. For A–F, concentration values were calculated from a standard curve and values resulting in a negative concentration are displayed as 0 and were recorded as 0 for statistical analyses.

Because of the large size of enzymes detected in *R. torques* supernatants and poorly successful attempts to express and purify recombinant forms of individual enzymes (not shown), we empirically assessed the collective catabolic abilities of the secretome. In accordance with a lack of predicted sulfatases, we did not observe activities of *R. torques* precipitated proteins on sulfated monosaccharides (Fig. S5). But, we did observe sulfatase activity in sonicated *B. thetaiotaomicron* cultures after exposure to keratan sulfate to induce these functions, including activities previously reported for this species (30) (Fig. S5). *R. torques* cleavage of the disaccharide *N*-acetyllactosamine (LacNAc), which presents a terminal, non-reducing β1,4-linked galactose, was observed (Fig. 4A; Fig. S6A). Interestingly, cleavage of lacto-*N*-biose (LNB), a disaccharide isomer of LacNAc with a β1,3 galactosidic linkage, was weaker, suggesting that *R. torques* is less proficient at removing terminal galactose when present as a β1,3 linkage (Fig. 4A; Fig. S6A, B, and H).

L-fucose was released efficiently from all four fucosylated substrates, including Lewis a, b, x, and y (Fig. 4B and C; Fig. S6C through F). Thus, *R. torques* enzymes have α-L-fucosidase activities on all relevant mucin glycan linkages (fucose-α1,2-galactose, fucose-α1,3-*N*-acetylglucosamine, fucose−α1,4-*N*-acetylglucosamine), with no apparent linkage preference (Fig. 4B and C). Enhanced activity on galactose-β1,4-*N*-acetylglucosamine (type 2 chain) vs. galactose-β1,3-*N*-acetylglucosamine (type 1 chain) is also apparent with these substrates, as more galactose was released from Lewis x and Lewis y than Lewis a or Lewis b (Fig. 4B and C). We tested activity on two additional terminal *O-*glycan epitopes, blood groups A and B (44). Strong activity was observed on blood group A, but not on blood group B (Fig. 4D; Fig. S7A through D).

We sought to understand whether monosaccharides were also released from the more complex mucin glycoproteins. Both L-fucose and galactose concentrations were significantly increased after 24-h incubation of *R. torques* supernatants with cMUC2 and PGM (Fig. 4E and F). After normalizing monosaccharide concentrations to mucin glycoprotein concentrations, less L-fucose and galactose were released from cMUC2 (L-fucose: 0.032 ± 0.005, galactose: 0.020 ± 0.002) than from PGM (L-fucose: 0.053 ± 0.012, *P* = 0.0027; galactose: 0.075 ± 0.023, *P* = 0.0001). Glycans in cMUC2 are more sulfated than those in PGM (19), which may inhibit access of *R. torques* due to its observed lack of sulfatase activities (Fig. S5). Interestingly, *R. torques* supernatants from cultures previously grown on gMO also released L-fucose and galactose from cMUC2 and PGM (Fig. S8A and B). This suggests that the previously observed decrease in mucin degradation (Fig. 2B, C, E, and F) from gMO-grown supernatants is not due to an inability to release these two monosaccharides. Collectively, we have demonstrated the presence of α-L-fucosidase and β-galactosidase activities in the *R. torques* secretome, active on free *O*-glycans and mucin glycoprotein domains.

### Digestion of mucin glycans during *R. torques* growth

**Fig 5:**
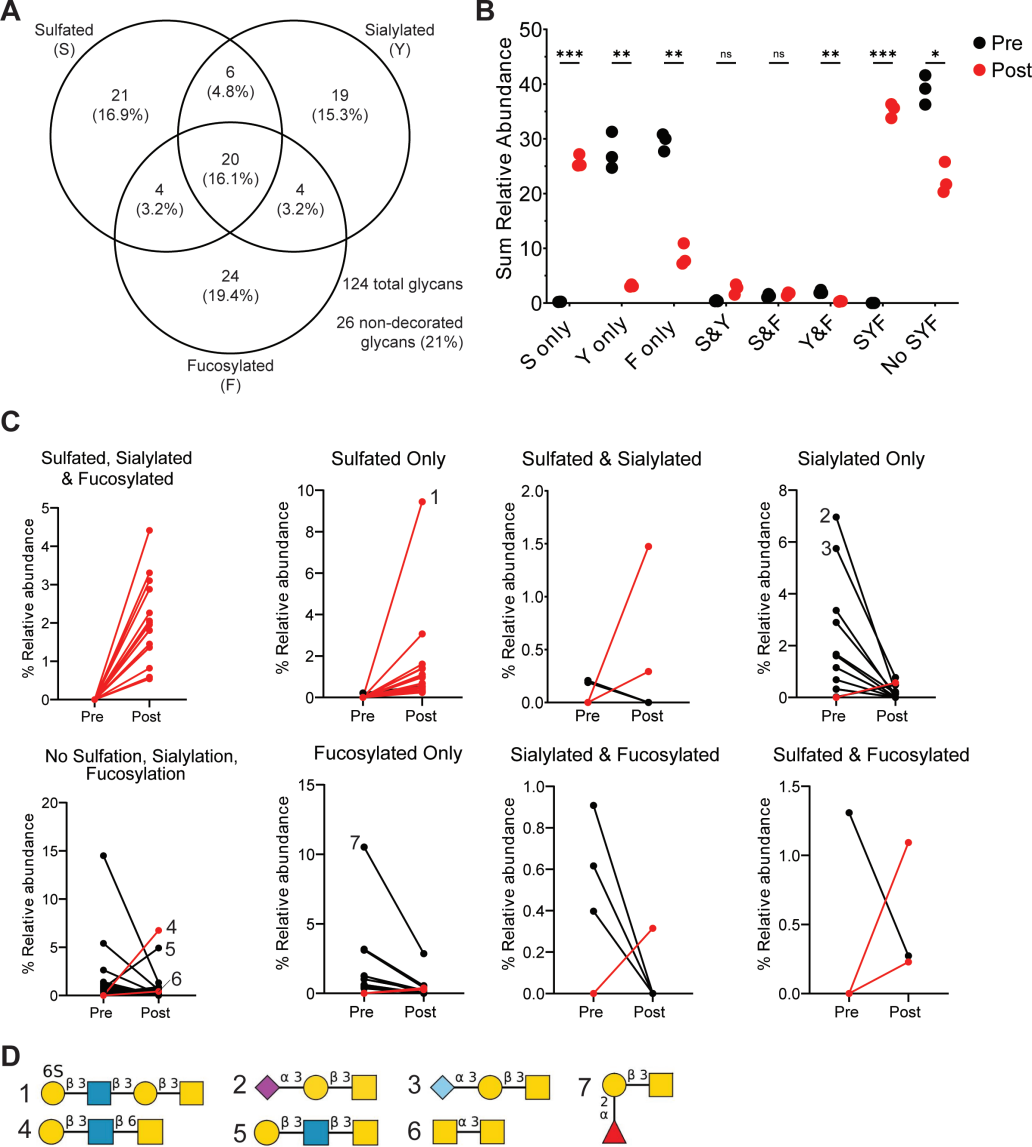
Fucosylated and sialylated glycans are degraded after growth of *R. torques* on cMUC2, but sulfated glycans accumulate. (A) Number and percentage of total glycans detected in cMUC2 pre- and/or post-growth of *R. torques* displaying indicated structural features by LC-MS/MS. (B) Sum relative abundance of glycans and inferred peeling reaction products pre- or post-*R. torques* growth on cMUC2 with respective structural features. Statistical analysis was performed with paired, two-tailed *t*-tests between pre- and post-growth samples for each structural feature category. *P < 0.05, **P < 0.01, ***P < 0.001. (C) Relative abundance of glycans detected that were retained on the mucin polypeptide backbone and were significantly different in pre- or post-growth samples of *R. torques* on cMUC2. Red lines indicate glycans that were not detected in any of the pre-growth samples but were detected in the post-growth samples. Each point represents the average relative abundance of three samples. (D) Putative structures of select detected glycans. Numbers refer to the corresponding plot in panel C. Structures generated with DrawGlycan (44). F, fucosylated; ns, not significant; S, sulfated; Y, sialylated.

We next sought to identify specific mucin glycan structures degraded during *R. torques* growth or structures recalcitrant to degradation, which could become available to other microbiota members. Residual glycoproteins were isolated from samples collected pre- and post-*R. torques* growth on cMUC2 or PGM, released from the polypeptide backbone by reductive β-elimination, and analyzed using LC-MS/MS. Because of the difficulty in separating free glycans from cultivated medium (data not shown), gMO and cMO were not analyzed. We first examined each glycan present pre- and/or post-growth, excluding peeling reaction products generated by reductive β-elimination (45), and measured the prevalence of three terminal capping features: sulfation, sialylation, and fucosylation (Fig. 5A). As expected (19), cMUC2 contained many sulfated and sialylated glycans (Fig. 5A). We also measured the mass-to-charge ratio, deduced structure (when possible), and calculated relative abundance to determine whether individual glycans, including putative peeling reaction products (Fig. S9), were degraded or accumulated.

The sum relative abundance of most glycans containing sulfate increased significantly after *R. torques* growth on cMUC2 (Fig. 5B). Glycans that were sulfated and sialylated or sulfated and fucosylated did not change in abundance, though these represent minorities of the total glycan abundance (Fig. 5B). In contrast, non-sulfated glycans that were fucosylated and/or sialylated, or glycans lacking all capping features, decreased in abundance after *R. torques* growth (Fig. 5B). This suggests strong sialidase and fucosidase activities, the latter of which is concordant with our supernatant activity (Fig. 4B through F).

Examining glycans with significantly different relative abundances*,* we noted that most glycans containing sulfate were undetected before growth and increased in abundance after growth (Fig. 5C; Fig. S9A,B and G). Notably, many newly detected sulfated glycans after *R. torques* growth were large (>1 kDa); these glycans were likely present originally as components of larger glycans exceeding detectable mass that were then partially degraded by *R. torques* to detectable size (Table S4). The accumulation of sulfated glycans was quite remarkable, with one glycan undetectable before growth representing >9% average relative abundance after growth (Fig. 5C and D, structure #1). Indeed, the accumulation of sulfated glycans after *R. torques* growth further suggests a lack of sulfatase activity, leading to their retention on mucin polypeptides.

Evidenced by their loss, sialylated and fucosylated glycans were apparently more degraded by *R. torques*. The majority of glycans significantly different in relative abundance containing these features were present in pre-growth samples but decreased in abundance after growth (Fig. 5C, Figure S9F, H, and D). Putative structures from the most highly abundant sialylated glycans suggest an ability of *R. torques* to remove both *N*-acetylneuraminic acid and *N*-glycolylneuraminic acid from select glycans (Fig. 5C and D, structures #2 and #3). The deduced structure of a highly abundant fucosylated glycan pre-growth that decreased in abundance post-growth suggests the presence of a fucose-α1,2-galactose activity (Fig. 5C and D, structure #7), which we previously observed on Lewis b and y (Fig. 4C).

Glycans lacking all three capping features overall decreased in abundance (Fig. 5B and C; Fig. S9C). Of note, the two most abundant glycans in this category post-growth present terminal galactose-β1,3-*N*-acetylglucosamine linkages (Fig. 5C and D, structures #4 and #5). This further supports a deficiency in *R. torques* enzymes that cleave this linkage, consistent with the weak activities noted above on Lewis a, Lewis b, and LNB (Fig. 4A through C). *N*-acetylgalactosamine-α1,3-*N*-acetylgalactosamine also accumulated, suggesting a lack of activity targeting this linkage (Fig. 5C and D, structure #6). This putative structure is known as core 5, a less commonly reported mucin core structure found in human meconium and intestinal cancer (46). It is also of note that 16 *N-*glycans were detected pre- and/or post-growth and were included in the above analyses (Table S4). Of these, only half were significantly different in abundance between pre-growth and post-growth samples. Overall, *N*-glycans decreased in abundance after growth, representing ~12% relative abundance pre-growth and ~6% relative abundance post-growth.

The relative levels of sulfation, sialylation, and fucosylation were also as expected for PGM, including high prevalence of fucosylation (19) (Fig. S10A). Similar to cMUC2, the sum relative abundance of fucosylated and/or sialylated glycans decreased, while sulfated glycans and glycans lacking these features increased in abundance post-growth (Fig. S10B). One glycan predicted to contain an internal *N*-acetylglucosamine-6S linkage was undetected pre-growth but represented >4% relative abundance of all glycans after growth (Fig. S10C and D, structure #1; Fig. S511D). Interestingly, a deduced structure lacking all three features decreased dramatically in abundance post-growth, suggesting an ability to release *N*-acetylglucosamine-α1,4-galactose from mucin glycans (Fig. S10C and D, structure#2; Fig. S11B). In contrast, galactose-β1,3-*N-*acetylgalactosamine (core 1) increased dramatically (from <1% to >20%) in abundance after growth, suggesting deficient activity against this structure (Fig. S10C and D, structure #3; Fig. S11B) in accordance with our data above (Fig. 4A). Most sialylated glycans were degraded, including one with a terminal *N*-acetylneuraminic acid-α2,6-*N*-acetylgalactosamine linkage (Fig. S10C and D, structure #4; Fig. S11F). Additionally, 12 *N-*glycans were identified in pre- and/or post-growth samples, 8 of which were significantly different in abundance between pre- and post-growth samples. *N-*glycans increased in abundance overall from ~1.4% relative abundance pre-growth to ~6.8% relative abundance post-growth.

While most fucosylated glycans decreased in abundance post-growth*,* the predicted structure fucose-α1,2-galactose-β1,3-*N*-acetylgalactosamine increased in abundance post-growth (Fig. S10C and D, structure #5; Fig. S11A), opposite of the trend observed for this glycan in cMUC2 (Fig. 5C and D). Key enzymes involved in degrading this substrate may not be expressed or active during growth on PGM. Alternatively, variation in glycan location and distribution throughout the mucin polypeptide backbone in PGM vs. cMUC2 may alter access of enzymes to these glycans, which we are unable to assess using this approach. Collectively, our data indicate that *R. torques* efficiently degrades many fucosylated and sialylated mucin glycans but largely fails to degrade sulfated ones or sulfated regions of larger glycans.

### *R. torques* enhances growth of *B. thetaiotaomicron* on porcine gastric mucin

Our data suggest that *R. torques* liberates monosaccharides and possibly longer *O*-glycan fragments from mucin glycans while leaving partially digested *O*-glycans bound to the mucin polypeptide backbone. We next sought to examine whether these products become accessible to other mucin-degraders. *B. thetaiotaomicron* degrades mucin *O-*glycans but grows poorly or not at all on mucin glycoproteins (Fig. 1A and B) (5, 26). Given the latter observation, we sought to understand interspecies interactions between *R. torques* and *B. thetaiotaomicron* with respect to utilization of PGM, the more readily available mucin glycoprotein substrate. Fortuitously, *R. torques* grows robustly (max *A*_600_ 0.87 ± 0.01 in 48 h) in a partially defined medium (DM) when PGM is present as the major carbohydrate source (Fig. S12). This medium is also suitable for growth of *B. thetaiotaomicron* and other *Bacteroides* (47, 48). As *B. thetaiotaomicron* grows comparatively poorly (max *A*_600_ 0.34 ± 0.01 in 48 h) by itself in DM-PGM medium (Fig. S12), we hypothesized that co-culture with *R. torques* would support enhanced *B. thetaiotaomicron* growth. While the total growth yield of *R. torques* is higher than *B. thetaiotaomicron* on PGM, *B. thetaiotaomicron* reaches its maximum growth faster (~9 h) than *R. torques* (~19 h), which may confer a competitive advantage for *B. thetaiotaomicron* accessing shared products in co-culture with *R. torques*. Furthermore, the exponential doubling time of *B. thetaiotaomicron* (1.07 h ± 0.04 h) on PGM was not significantly different than that of *R. torques* (1.07 h ± 0.15 h), suggesting that *B. thetaiotaomicron* efficiently utilizes the fewer components of PGM that it can access.

**Fig 6:**
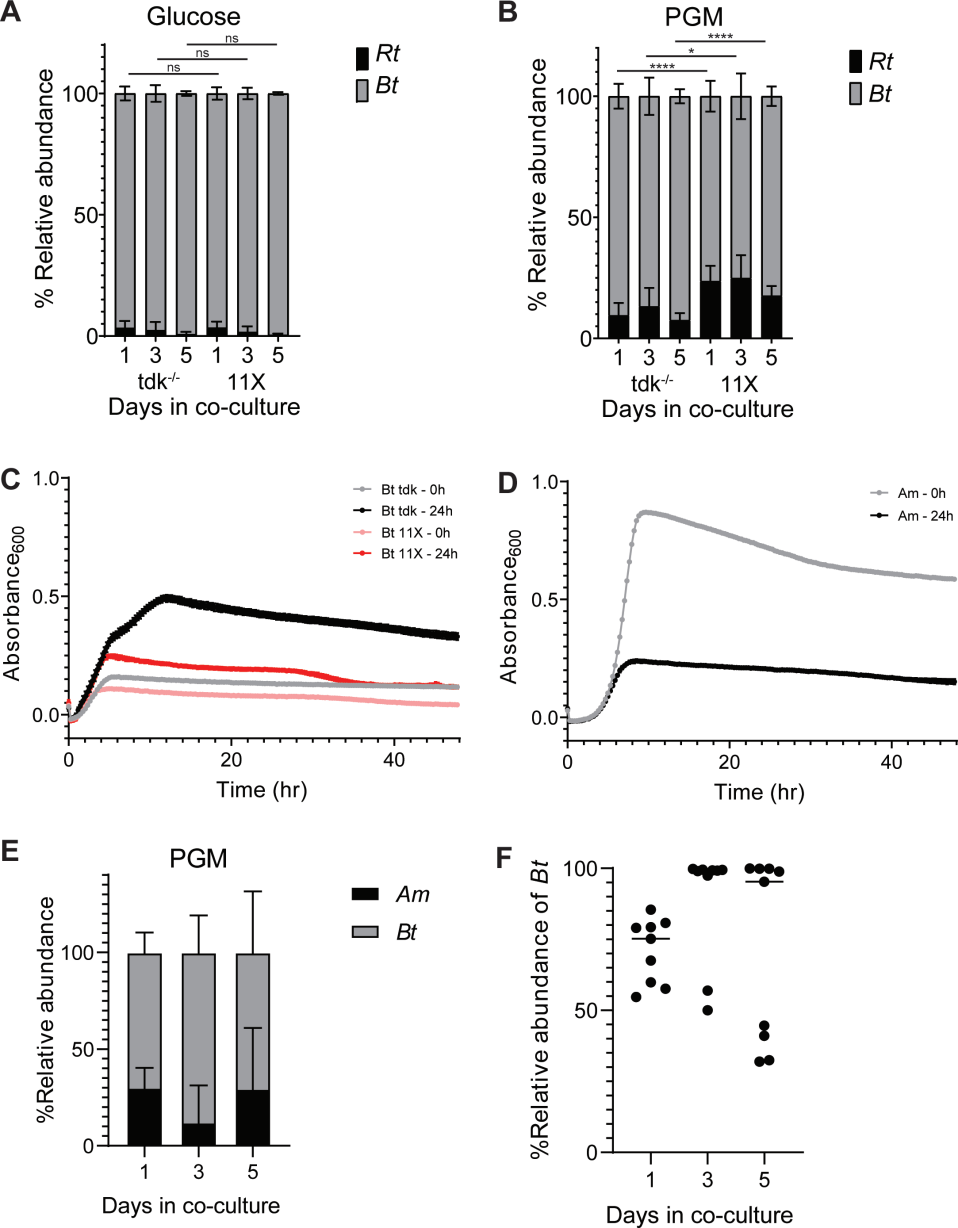
*R. torques* generates monosaccharide and *O-*glycans products from PGM that *Bacteroides thetaiotaomicron* can utilize. (A and B) Relative abundance of *R. torques* and *B. thetaiotaomicron tdk*^−/−^ or 11× mutant in co-culture grown in partially DM and glucose (A, 5 mg/mL) or PGM (B, 10 mg/mL) calculated by quantitative PCR. Statistics were analyzed with two-tailed, unpaired *t*-tests comparing relative abundance of *R. torques* vs. *B. thetaiotaomicron tdk*^−/−^ co-culture to relative abundances in *R. torques* vs. *B. thetaiotaomicron* 11× mutant co-culture at each respective timepoint. *P < 0.05, ****P < 0.0001; *n* = 9. (C and D) Growth of *B. thetaiotaomicron* (*tdk*^−/−^, 11× mutant, C) and *A. muciniphila* (D) on PGM pre-digested by *R. torques* supernatant for 0 or 24 h. *B. thetaiotaomicron* was grown in *Bacteroides* minimal medium, and *A. muciniphila* was grown in chopped meat medium. *n* = 3; points represent mean ± standard deviation. (E) Relative abundance of *A. muciniphila* and *B. thetaiotaomicron* in co-culture grown in partially-defined DM medium with PGM (5 mg/mL). (F) Relative abundance of *B. thetaiotaomicron* in each experiment shown in panel E. DM, defined medium; ns, not significant.

To test if *R. torques* supports *B. thetaiotaomicron* growth on PGM, both species were co-cultured in DM with glucose or PGM and were passaged daily for 5 days with a 1:20 dilution factor. Measured by relative abundance, *B. thetaiotaomicron tdk*^−/−^ [a thymidine kinase-deficient parent strain in which allelic exchange mutants are generated (49)] dominated in co-culture on glucose, representing >91% of the community (Fig. 6A). Surprisingly, *B. thetaiotaomicron tdk*^−/−^ also dominated on PGM, consistently representing >76% relative abundance through day 5 (Fig. 6B). The dominance of *B. thetaiotaomicron* in co-culture with *R. torques* on PGM, despite its comparatively poor growth in monoculture, suggests that *R. torques* liberates products from PGM that *B. thetaiotaomicron* can access.

*B. thetaiotaomicron* is genetically tractable, and many genes in this species have been associated with *O*-glycan degradation (26, 30, 50). Some previously generated mutant strains are deficient at using *O*-glycans *in vitro* or *in vivo* (15, 30). To test whether the consumption of released mucin *O-*glycans—as opposed to liberated monosaccharides—was critical for *B. thetaiotaomicron* to be supported by *R. torques* on PGM, we co-cultured *R. torques* with a *B. thetaiotaomicron* mutant lacking 93 genes spanning 11 polysaccharide utilization loci (mutant called *B. thetaiotaomicron* “11×”) (15). This mutant has a large growth defect on gMOs and *N*-acetylgalactosamine but not on other mucin monosaccharides (Fig. S13A through F). Compared to co-cultures with the parent *B. thetaiotaomicron tdk*^−/−^, the 11× mutant strain has no defect in co-culture with *R. torques* on glucose but shows a significant defect on PGM (Fig. 6A and B). This suggests that *B. thetaiotaomicron* relies on its ability to utilize *O-*glycans and/or free *N*-acetylgalactosamine through the 11 missing systems to access some PGM products released by *R. torques*.

To further assess the contribution of *O-*glycan utilization to *B. thetaiotaomicron* persistence in co-culture with *R. torques* on PGM*,* we incubated PGM with *R. torques* supernatants. These substrates were treated, including dialysis (1 kDa cutoff) to remove monosaccharides (Fig. S13G and H), and used as a growth substrate for *B. thetaiotaomicron*. In a medium supplemented with the 0-h control digest, *B. thetaiotaomicron* grew (max *A*_600_ 0.160 ± 0.004 in 48 h) similar to untreated PGM (max *A*_600_ 0.212 ± 0.008 in 48 h) (Fig. 1A and 6C). Total *B. thetaiotaomicron* growth increased dramatically on the *R. torques* supernatant 24-h digested PGM (Fig. 6C). This further suggests that *R. torques* releases products from PGM that *B. thetaiotaomicron* can use for growth but cannot access alone. Since this substrate was dialyzed to remove monosaccharides, *R. torques* supernatants must release some *O-*glycan fragments in addition to free monosaccharides. While the growth of the *B. thetaiotaomicron* 11× mutant increased on the 24-h *R*. *torques* PGM digest relative to the 0-h control digest, the average maximum absorbance values of the *B. thetaiotaomicron* 11× mutant after growth on the 24-h digest (0.247 ± 0.008) were only 50.3% of the average maximum absorbances of *B. thetaiotaomicron tdk*^−/−^ (0.492 ± 0.013) (Fig. 6C). This further suggests that the products released by *R. torques* from PGM include *O-*glycans, as the 11× mutant has a strong growth defect on gMO (Fig. S13A).

While our results support a model of cross-feeding between *R. torques* and *B. thetaiotaomicron*, they suggest a different relationship exists between *R. torques* and *A. muciniphila*. Like *R. torques*, *A. muciniphila* grows well on undigested PGM (Fig. 1). However, *A. muciniphila* displayed decreased total growth on *R. torques* digested PGM compared to undigested PGM (Fig. 6D), suggesting *R. torques* products released from PGM are an insufficient growth substrate for *A. muciniphila*. This suggests that each species uses different mechanisms to release unique products and that the products released from PGM by *R. torques* are not an optimal nutrient source for *A. muciniphila*, consistent with its weaker growth on chemically released mucin glycans compared to their parent glycoproteins (Fig. 1A through D). Despite the inability of *A. muciniphila* to benefit from *R. torques* mucin degradation, *A. muciniphila* supported *B. thetaiotaomicron* in co-culture on PGM (Fig. 6E). Interestingly, while *B. thetaiotaomicron* persisted in all co-culture experiments, representing at least 30% relative abundance, in five of nine co-cultures, *B. thetaiotaomicron* was very dominant (>95% relative abundance) at day 5 (Fig. 6F). These results further suggest that like *R. torques*, *A. muciniphila* is likely a keystone mucin degrader, though there are likely key mechanistic differences in how these two species degrade mucins that prevent nutrient sharing between them.

## DISCUSSION

Previous reports have connected the activity of mucin-degrading bacteria to mucus defects *in vivo* and subsequent disease development (5, 12–15). Thus, it is important to define bacterial mucin-degrading mechanisms and investigate their impacts on the microbiota and host health. Here, we advance the understanding of the mucin-degrading mechanisms deployed by *R. torques*. Of note, *R. torques* utilizes mucin glycoprotein domains and released *O*-glycans from the stomach and the colon, degrades these substrates with largely constitutively secreted enzymes, and releases products from intact gastric mucins that become available to *B. thetaiotaomicron*. The ability of *R. torques* to cross-feed products released from intact mucin glycoproteins to *B. thetaiotaomicron* supports the hypothesis that *R. torques* is a keystone mucin degrader. This definition is in line with similarly defined roles for other gastrointestinal *Ruminococci* (51) [*R. bromii* (52) and *R. flavefaciens* (53)] that degrade resistant starch and cellulose, respectively.

We identified features of the *R. torques* mucin-degrading mechanism that foster understanding of its role within the gut community. Recent studies have investigated bacterial degradation of colonic mucins (30–33), while previous studies predominantly examined gastric mucin. The data we present here underscore the importance of using multiple sources (gastric vs. colonic) and forms (intact mucin domains or released, free *O*-glycans) of mucins to enhance knowledge of how bacteria access nutrition from these complex substrates. The broad range of mucin substrates utilized by *R. torques* led us to hypothesize it would have a strong ability to influence the gut community.

The general lack of transcriptional activation of putative CAZymes in response to *O-*glycans by *R. torques* differs from what has been observed in *B. thetaiotaomicron* and other *Bacteroides* spp. (5, 26, 42, 54, 55). However, the repression of *R. torques* mucin degradation in the presence of gMO suggests a potential negative feedback loop in which released glycans from mucin glycoproteins suppress these systems in *R. torques* through post-transcriptional mechanisms. Such a system in *R. torques* may be fortuitously protective for the host by preventing excessive mucus erosion when free *O-*glycans are in excess, which may be disrupted in cases of disease. Our findings also suggest that these enzymes are constitutively expressed by *R. torques* during growth in non-mucin substrates. Like *A. muciniphila* (5), the growth profile of *R. torques* is predominantly limited to mucin components. Thus, constitutive expression of mucin-degrading enzymes may promote *R. torques* fitness despite the high energetic cost because they are almost always required to access its narrow mucin nutrient niche.

The predominantly extracellular *R. torques* mucin degradation is similar to that observed in other species (23) including *Bifidobacterium bifidum* (56–58) and *R. gnavus* (59), suggesting this may be a common feature of Gram-positive mucin degraders. In contrast, Gram-negative mucin degraders, including *A. muciniphila* (40) and *B. thetaiotaomicron* (30), are thought to import mucin glycoproteins or their fragments and degrade them in the periplasm. It has been previously proposed that the location of mucin degradation has significant implications for the effects of these activities on the community. Indeed, our experiments suggest that *R. torques* releases monosaccharides and oligosaccharides from intact mucin glycoprotein but leaves the majority of sulfated glycans attached to the polypeptide backbone, and that these products become available for other species less equipped to access intact mucin. However, the ability of *A. muciniphila* to support *B. thetaiotaomicron* in co-culture on PGM suggests that mucin degraders with intracellular degradation mechanisms can still support other species, further underscoring the importance of *in vitro* characterization of interactions between different species.

Understanding the role of *R. torques* and other keystone degraders within the gut community may provide insight into the impairment of the colonic mucus layer as a potential first step in the progression of spontaneous inflammation in the colon. Causal roles for mucin-degrading bacteria are emerging in IBD models (13, 60, 61). These roles may be filled by multiple different species, including keystone mucin-degrading bacteria and corresponding secondary degraders, that perform interchangeable functional roles and possibly work synergistically (e.g., if *B. thetaiotaomicron* consumes *O-*glycan fragments that might inhibit *R. torques* degradative enzymes). Here, *R. torques* and *B. thetaiotaomicron* are demonstrated to be one pair of species engaging in primary mucin degradation and cross-feeding *in vitro*. Further studies are needed to assess whether this relationship exists *in vivo*, whether the presence of these two species together contributes to worsened disease in susceptible hosts, and if the activity of this bacteria pair further influences other members of the gut bacterial community. Identifying additional keystone mucin degraders and their benefactors may illuminate novel diagnostic approaches to detect aberrant communities and interspecies interactions which, in a susceptible host, may promote disease. Further characterization of molecular mechanisms of identified keystone mucin degraders may also open potential avenues for development of novel therapeutic targets to block IBD development.

## MATERIALS AND METHODS

### Preparation of mucin substrates

PGM (American Laboratories) was purified as previously described using ethanol precipitation (22). Gastric mucin *O*-glycans (gMO) were generated from porcine gastric mucin (type III, Sigma), using a previously described method (62). Colonic mucin glycoprotein (cMUC2) was purified from porcine colons (anus through ~2 feet into the colon) obtained from the Michigan State University Meat Laboratory. cMUC2 was purified using previously reported procedures (30, 63). cMO were purified from cMUC2 using the same method used to generate gMO, except they were not subjected to diethylaminoethyl anion exchange chromatography due to the high abundance of sulfated glycans in colonic mucus. Final stocks of cMUC2 and PGM were resuspended in phosphate-buffered saline (PBS) pH 7.4 to improve solubility.

### Bacterial strains and mucin growth curves

All bacterial cultures were grown in an anaerobic chamber (Coy Labs; 85% N_2_, 10% H_2_, 5% CO_2_) at 37°C. *R. torques* VIII-239 was grown overnight in a previously described modified YCFA medium (37, 52). *B. thetaiotaomicron tdk*^−/−^ strain [a thymidine kinase-deficient mutant in *B. thetaiotaomicron* VPI-5482 used to generate allelic exchange mutants (49)] was used throughout this study and was grown overnight in tryptone yeast extract glucose (TYG) medium (64). *A. muciniphila* DMS 22959 was grown overnight in a custom chopped meat medium (48). For growth curves, cells from rich medium culture were pelleted, washed in carbohydrate-free media [*R. torques*: YCFA no glucose, *A. muciniphila*: chopped meat no sugars; *B. thetaiotaomicron*: *Bacteroides* minimal media no glucose (26)], and diluted (1:40), and 100 µL of washed cells was added to 100 µL of growth substrates in 96-well plates (Costar). Growth substrates were tested at the following final concentrations: glucose/*N*-acetylglucosamine (5 mg/mL), mucin *O*-glycans (gMO, 10 mg/mL), PGM (10 mg/mL), colonic *O*-glycans (cMO, 5 mg/mL), and cMUC2 (2.5 mg/mL). Growth was measured by optical density at 600 nm (OD_600_) at ~10-min intervals using a microplate stacker (BioTek BioStack) and microplate reader (BioTek PowerWave). Growth curve OD_600_ values were normalized by subtracting average baseline readings (from the first three OD readings) from all readings to correct for the background OD_600_ of substrates.

### Gel electrophoresis and periodic acid-Schiff staining

*R. torques* cultures were grown overnight in YCFA with glucose (5 mg/mL) or gMO (10 mg/mL). Culture samples were added directly to reaction mixtures, and supernatants were collected by centrifugation for 2 min at 8,000 rpm and added 1:1 to cMUC2 (2.5 mg/mL) or PGM (10 mg/mL) and incubated anaerobically for 2 days. Samples were collected, diluted 1:4 in Laemmli sample buffer (Bio-Rad), and heated for 10 min at 98°C. Samples were loaded onto 4%–12% Bis-Tris gradient gels (Thermo Fisher) unless otherwise noted and run for 60 min at 180 V. Gels were stained with periodic acid-Schiff stain as previously described (65) and imaged using Bio-Rad GelDoc Imaging System. The intensity of mucin bands was quantified by densitometry with ImageJ, and intensity from a blank lane in each gel was subtracted from all other lanes in the respective gel to control for background staining across gels. The percent degradation in various conditions was calculated relative to controls lacking *R. torques* culture or supernatant.

### RNA sequencing of *R. torques* on glucose vs. gMO

*R. torques* was grown in 5 mg/mL glucose or 10 mg/mL gMO using the protocols above for growth curves. Washed cells from each overnight replicate were added to the plate in 12 replicate wells to increase biomass. Cultures were collected during mid-log phase (OD_600_ ~0.7 to 0.9), pooling the 12 replicate wells per sample. RNA was extracted as previously described for stool or cecal contents, except samples were precipitated with isopropanol and the DNase source (NEB) (66). rRNA was depleted from the samples using the MICROBExpress Bacterial mRNA Enrichment Kit (Thermo Fisher). RNA was submitted to the University of Michigan Advanced Genomics Core for library preparation and RNA-sequencing (Illumina NovaSeq platform, 150-bp read length, paired end).

Reads were analyzed using previously reported methodology (47). Briefly, Trimmomatic v.0.39 was used to filter reads for quality. Reads were aligned to the *R. torques* genome using BowTie2 v.2.3.5.1. Reads mapping to gene features were counted using htseq-count (release_0.11.1). Differential expression analysis was performed with the edgeR v.3.34.0 package in R v.4.0.2 (with the aid of Rstudio v.1.3.1093). Library normalization was performed using the trimmed mean of *M* values method. Integrated Genome Viewer was used to visualize coverage data.

### Proteomics

*R. torques* overnight cultures in YCFA with glucose were grown and centrifuged at 7,830 rpm for 2 min. Samples were filtered where noted using a 0.22 µM polyvinylidene difluoride (PVDF) syringe filter. Proteins were precipitated using a pyrogallol red methanol molybdate (PRMM) solution. PRMM was made with 20% methanol, 0.05 mM pyrogallol red, 0.16 mM sodium molybdate, 1 mM sodium oxalate, 50 mM succinic acid, and adjusted to pH 2. PRMM was added 1:1 with isolated supernatants, and pH was adjusted to pH of 3. Samples were incubated for 2 h at room temperature and then overnight at 4°C. Samples were then centrifuged for 60 min at 10,000 × *g* at 4°C, and pellets were washed with cold acetone. Centrifugation and washing were repeated once, and pellets were dried and resuspended in 8 M urea with 50 mM HEPES at pH 8.

Samples were submitted to the Proteomics Resource Facility (PRF) and were analyzed using high-resolution LC-MS/MS analysis per the protocol optimized by the PRF and previously described (47). The following modifications were made from the previous protocol: cysteines were alkylated with 65 mM 2-chloroacetamide; trypsin digestion was performed after diluting urea to <1.2 M and using 0.5 µg trypsin (Promega); and processed peptides were dissolved in 20 µL of buffer A. Peptides were resolved over a period of 90 min (2%–25% buffer B in 45 min, 25%–40% in 5 min, and 40%–90% in 5 min, then held at 90% buffer B for 5 min and equilibration with buffer A for 30 min). Proteins were identified by searching the generated tandem mass spectrometry data against a database of all protein sequences present in *R. torques* VIII-239. Parameters used were the same as mentioned previously, except for the use of a 0.1-kDa fragment tolerance. Relative abundance of proteins was calculated based on MS1 abundances.

### *R. torques* supernatant enzyme activity assays on model glycans

*R. torques* cultures (40 mL) were spun at 7,200 × *g* for 10 min, repeated as necessary to pellet cells, and 60 mL of 4 M ammonium sulfate was added to the supernatant. Samples were incubated for 30 min at room temperature and centrifuged at 18,000 × *g* for 30 min at 4°C, and the precipitate was resuspended in 1 mL of PBS. Samples were dialyzed overnight into PBS in 3.5-kDa cutoff dialysis tubing at 4°C. Reaction mixtures contained 2 mM [Lewis a, b, x, and y (Dextra); blood groups A and B (Elicityl)] or 5 mM [LacNAc and LNB (CarboSynth/Biosynth)] substrate; 50 mM phosphate buffer, pH 7; and 10 µL precipitated protein solution. Sulfated sugar (Dextra) reactions contained 10 mM 2-(N-morpholino)ethanesulfonic acid (MES) buffer with 5 mM CaCl_2_, pH 6.5. *B. thetaiotaomicron* was grown overnight in *Bacteroides* minimal medium with 5 mg/mL final concentration of glucose; cells were collected, washed, and resuspended in *Bacteroides* minimal medium with 5 mg/mL keratan sulfate and incubated for 1 h prior to sonication (30% amplitude; 1 s on, 9 s off; 30-s total). A 10 μL sample of this preparation was added to the same reaction composition as a positive control for sulfatase activity. Reactions were incubated overnight at 37°C and stored at −20°C until analysis.

Samples were diluted 1:10 in water and were analyzed by high performance anion exchange chromatography with pulsed amperometric detection (Dionex ICS-6000 HPIC System, Thermo Scientific) using a CarboPac PA100 column (Thermo Scientific). The following buffers were used: buffer A (100 mM NaOH), buffer B (100 mM NaOH and 500 mM sodium acetate), and buffer C (water). The following method sequence was utilized for all samples with a constant flow rate of 1 mL/min: 15 min pre-equilibration with 90% buffer C and 10% buffer A; 20 min with 90% buffer C and 10% buffer A; 20 min gradient from 90% buffer C, 10% buffer A to 100% buffer A; 20 min gradient from 100% buffer A to 100% buffer B; 10 min with 100% buffer B; 10 min with 90% buffer C and 10% buffer A. Chromatograms were analyzed and peaks were quantified in Chromeleon Chromatography Data System Software (Thermo Fisher) using the basic quantification method. Standard curves were created to calculate concentrations of respective sugars from peak areas.

Sulfated glycan reactions were analyzed using thin-layer chromatography (TLC). Each reaction was spotted (3 µL) onto a silica plate (TLC silica gel 60, MilliporeSigma), placed in 100 mL of running buffer of butanol:water:acetic acid (2:1:1), and run to the end of the plate. The plate was dried and submerged in developer (2 g diphenylamine, 100 mL ethyl acetate, 2 mL aniline, 10 mL 85% H_3_PO_3_, and 1 mL 37.5% HCl) for ~30 s and dried again. Sugars were visualized by heating the plate over a flame.

### Analysis of released monosaccharides from mucin glycoprotein substrates

*R. torques* cultures were grown overnight in YCFA with glucose, and aliquots were removed and centrifuged at 8,000 rpm for 2 min. Supernatants were isolated and incubated with mucin substrates PGM (10 mg/mL final concentration) or cMUC2 (2.5 mg/mL final concentration) 1:1 by volume. Samples were incubated anaerobically at 37°C and were stored immediately (0-h control) or incubated for 24 h. Samples were analyzed using the L-Arabinose/D-Galactose Assay Kit (Megazyme) and the L-Fucose Assay Kit (Megazyme) per kit protocols for the microplate assay format. Baseline absorbance for each sample and the absorbance from the corresponding negative control were subtracted from sample absorbance readings, and concentrations were calculated from a standard curve for each corresponding monosaccharide.

### Bioinformatic analysis of putative mucin-degrading enzymes

The presence of potential signal sequences to indicate secreted proteins in *R. torques* for transcriptomic/proteomic analyses was identified using SignalP v.5.0 (DTU Health Tech). Proteins identified as top hits from transcriptomic or proteomic data sets lacking a functional annotation (i.e., annotated as hypothetical proteins) were analyzed for putative functions using National Center for Biotechnology Information Protein BLAST. Domain analysis of putative mucus-degrading enzymes was performed using CAZy (43). Graphics representing domain architecture were generated using Illustrator for Biological Sequences (67). *R. torques* VIII-239 amino acid sequences were analyzed using SulfAtlas HMM using default settings and cutoffs (high prediction: score >300, low prediction: 200 < score <300 and coverage >80%, probable fragment: 100 < score <300 and coverage <80%) (68).

### LC-MS/MS analysis of mucin glycans before and after *R. torques* growth

*R. torques* was grown on cMUC2 and PGM using the same growth methods described above, and samples were collected immediately after inoculating cultures (pre-growth) and after cultures reached stationary phase (post-growth). Glycans from the samples were isolated by dot blot using a PVDF membrane and were analyzed using carbon-column liquid chromatograph-electrospray ionization tandem mass spectrometry, as previously described (32, 69).

### Growth of bacterial strains on *R. torques* supernatant digests of PGM

*R. torques* was grown overnight in YCFA with glucose and was centrifuged for 10 min at 7,197 g. The supernatant was collected and added 1:1 by volume to 20 mg/mL PGM. A sample was collected immediately as a control (0 h), and another sample was incubated anaerobically at 37°C for 24 h. Upon collection, samples were boiled for 10 min, then centrifuged at 7,197 × *g* for 10 min. The supernatant was collected and dialyzed (1-kDa cutoff) against 4 L water, changing the water three times. Samples were then lyophilized, resuspended at 20 mg/mL in PBS, and autoclaved with a 5 min sterilization cycle time. Growth experiments were performed as previously described, except strains were grown in partially DM (47, 48).

### Co-culture of *R. torques* and *B. thetaiotaomicron* on PGM

Overnight cultures of *R. torques* (YCFA with glucose), *B. thetaiotaomicron* (TYG), or *A. muciniphila* (chopped meat) were grown. One milliliter was taken from each culture, centrifuged for 2 min at 8,000 rpm, and washed in 2× DM. Centrifugation was repeated and cultures were resuspended in 2× DM. Respective cultures were added in a 1:1 vol ratio (10 µL of each species into each two strain co-culture), except in one triplicate set of *R. torques* and *B. thetaiotaomicron* co-cultures which were added in a 3:1 ratio (15 µL *R*. *torques* and 5 µL *B. thetaiotaomicron*) to a 96-well plate with 80 µL of 2× DM and 100 µL of substrate (5 mg/mL final glucose or 10 mg/mL final PGM). Co-cultures were grown anaerobically at 37°C and were passaged every 24 h. Samples were collected and frozen at −20°C until DNA was extracted using the DNeasy Blood and Tissue Kit (Qiagen) according to the following protocol: pre-treatment for Gram-positive bacteria, followed by the following protocol: purification of total DNA from animal tissues spin-column as described. DNA was quantified using a NanoDrop spectrophotometer (Thermo Scientific) and diluted to 5 ng/µL. Quantitative PCR (qPCR) reactions were performed as previously described (47), using *B. thetaiotaomicron* (BT1927F: 5-TCGCCTTTTTCAGATCAGTAGTTGG-3 and BT1927R: 5-ACGAAAATGGAGTTGAATGGAATAAGTT-3)

r *R. torques* (RT1159F: 5-CCGTTCCTGACAACATTAG-3; RT1159R: 5-GCCGATTCTCTTTCCTATATC-3) primers for *R. torques* and *B. thetaiotaomicron* co-cultures or previously generated qPCR primers (5) for *A. muciniphila* and *B. thetaiotaomicron* co-cultures. Corresponding standard curves were generated from purified genomic DNA from each respective species.

## Data Availability

RNA-sequencing data are deposited to the National Center for Biotechnology Information Gene Expression Omnibus under accession number GSE262875. LC-MS/MS data from the *R. torques* proteomics experiment are deposited in the Proteomics Identification Database under accession number PXD051107. Raw LC-MS/MS data from mucin glycans before and after growth of *R. torques* are deposited in GlycoPOST under accession number GPST000413.

## SUPPLEMENTAL MATERIAL

Supplemental Figures mbio.00039-24-s0001.pdf Figures S1 to S13.

Table S1 mbio.00039-24-s0002.xlsx RNA-sequencing gMO vs. glucose fold change data, functional annotations of differentially expressed genes, and RPKM in glucose and gMO.

Table S2 mbio.00039-24-s0003.xlsx Relative abundance of highly expressed proteins in the *R. torques* supernatant.

Table S3 mbio.00039-24-s0004.xlsx Top hits from proteomics and transcriptomics data sets.

Table S4 mbio.00039-24-s0005.xlsx Composition and putative structures of glycans.

Table S5 mbio.00039-24-s0006.xlsx Composition and putative structures of glycans.

ASM does not own the copyrights to Supplemental Material that may be linked to, or accessed through, an article. The authors have granted ASM a non-exclusive, world-wide license to publish the Supplemental Material files. Please contact the corresponding author directly for reuse.

## References

[1] Martens EC, Neumann M, Desai MS. 2018. Interactions of commensal and pathogenic microorganisms with the intestinal mucosal barrier. Nat Rev Microbiol 16:457–470. doi:10.1038/s41579-018-0036-x 29904082

[2] Earle KA, Billings G, Sigal M, Lichtman JS, Hansson GC, Elias JE, Amieva MR, Huang KC, Sonnenburg JL. 2015. Quantitative imaging of gut microbiota spatial organization. Cell Host Microbe 18:478–488. doi:10.1016/j.chom.2015.09.002 26439864 PMC4628835

[3] Schroeder BO, Birchenough GMH, Ståhlman M, Arike L, Johansson MEV, Hansson GC, Bäckhed F. 2018. Bifidobacteria or fiber protects against diet-induced microbiota-mediated colonic mucus deterioration. Cell Host Microbe 23:27–40. doi:10.1016/j.chom.2017.11.004 29276171 PMC5764785

[4] Sonnenburg JL, Xu J, Leip DD, Chen C-H, Westover BP, Weatherford J, Buhler JD, Gordon JI. 2005. Glycan foraging in vivo by an intestine-adapted bacterial symbiont. Science 307:1955–1959. doi:10.1126/science.1109051 15790854

[5] Desai MS, Seekatz AM, Koropatkin NM, Kamada N, Hickey CA, Wolter M, Pudlo NA, Kitamoto S, Terrapon N, Muller A, Young VB, Henrissat B, Wilmes P, Stappenbeck TS, Núñez G, Martens EC. 2016. A dietary fiber-deprived gut microbiota degrades the colonic mucus barrier and enhances pathogen susceptibility. Cell 167:1339–1353. doi:10.1016/j.cell.2016.10.043 27863247 PMC5131798

[6] Everard A, Belzer C, Geurts L, Ouwerkerk JP, Druart C, Bindels LB, Guiot Y, Derrien M, Muccioli GG, Delzenne NM, de Vos WM, Cani PD. 2013. Cross-talk between Akkermansia muciniphila and intestinal epithelium controls diet-induced obesity. Proc Natl Acad Sci U S A 110:9066–9071. doi:10.1073/pnas.1219451110 23671105 PMC3670398

[7] Shin N-R, Lee J-C, Lee H-Y, Kim M-S, Whon TW, Lee M-S, Bae J-W. 2014. An increase in the Akkermansia spp. population induced by metformin treatment improves glucose homeostasis in diet-induced obese mice. Gut 63:727–735. doi:10.1136/gutjnl-2012-303839 23804561

[8] Derrien M, Vaughan EE, Plugge CM, de Vos WM. 2004. Akkermansia muciniphila gen. nov., sp. nov., a human intestinal mucin-degrading bacterium. Int J Syst Evol Microbiol 54:1469–1476. doi:10.1099/ijs.0.02873-0 15388697

[9] Zocco MA, Ainora ME, Gasbarrini G, Gasbarrini A. 2007. Bacteroides thetaiotaomicron in the gut: molecular aspects of their interaction. Dig Liver Dis 39:707–712. doi:10.1016/j.dld.2007.04.003 17602905

[10] Porter NT, Larsbrink J. 2022. Investigation and alteration of organic acid synthesis pathways in the mammalian gut symbiont Bacteroides thetaiotaomicron. Microbiol Spectr 10:e0231221. doi:10.1128/spectrum.02312-21 35196806 PMC8865466

[11] Qin J, Li R, Raes J, Arumugam M, Burgdorf KS, Manichanh C, Nielsen T, Pons N, Levenez F, Yamada T, et al.. 2010. A human gut microbial gene catalogue established by metagenomic sequencing. Nature 464:59–65. doi:10.1038/nature08821 20203603 PMC3779803

[12] Neumann M, Steimle A, Grant ET, Wolter M, Parrish A, Willieme S, Brenner D, Martens EC, Desai MS. 2021. Deprivation of dietary fiber in specific-pathogen-free mice promotes susceptibility to the intestinal mucosal pathogen Citrobacter rodentium. Gut Microbes 13:1966263. doi:10.1080/19490976.2021.1966263 34530674 PMC8451455

[13] Pereira GV, Boudaud M, Wolter M, Alexander C, De Sciscio A, Grant ET, Trindade BC, Pudlo NA, Singh S, Campbell A, Shan M, Zhang L, Yang Q, Willieme S, Kim K, Denike-Duval T, Fuentes J, Bleich A, Schmidt TM, Kennedy L, Lyssiotis CA, Chen GY, Eaton KA, Desai MS, Martens EC. 2024. Opposing diet, microbiome, and metabolite mechanisms regulate inflammatory bowel disease in a genetically susceptible host. Cell Host Microbe 32:527–542. doi:10.1016/j.chom.2024.03.001 38513656 PMC11064055

[14] Parrish A, Boudaud M, Grant ET, Willieme S, Neumann M, Wolter M, Craig SZ, De Sciscio A, Cosma A, Hunewald O, Ollert M, Desai MS. 2023. Akkermansia muciniphila exacerbates food allergy in fibre-deprived mice. Nat Microbiol 8:1863–1879. doi:10.1038/s41564-023-01464-1 37696941 PMC10522492

[15] Hayase E, Hayase T, Jamal MA, Miyama T, Chang C-C, Ortega MR, Ahmed SS, Karmouch JL, Sanchez CA, Brown AN, et al.. 2022. Mucus-degrading bacteroides link carbapenems to aggravated graft-versus-host disease. Cell 185:3705–3719. doi:10.1016/j.cell.2022.09.007 36179667 PMC9542352

[16] Johansson MEV, Sjövall H, Hansson GC. 2013. The gastrointestinal mucus system in health and disease. Nat Rev Gastroenterol Hepatol 10:352–361. doi:10.1038/nrgastro.2013.35 23478383 PMC3758667

[17] Recktenwald CV, Hansson GC. 2016. The reduction-insensitive bonds of the MUC2 mucin are isopeptide bonds. J Biol Chem 291:13580–13590. doi:10.1074/jbc.M116.726406 27129250 PMC4919444

[18] Johansson MEV, Ambort D, Pelaseyed T, Schütte A, Gustafsson JK, Ermund A, Subramani DB, Holmén-Larsson JM, Thomsson KA, Bergström JH, van der Post S, Rodriguez-Piñeiro AM, Sjövall H, Bäckström M, Hansson GC. 2011. Composition and functional role of the mucus layers in the intestine. Cell Mol Life Sci 68:3635–3641. doi:10.1007/s00018-011-0822-3 21947475 PMC11114784

[19] Robbe C, Capon C, Coddeville B, Michalski J-C. 2004. Structural diversity and specific distribution of O-Glycans in normal human Mucins along the intestinal tract. Biochem J 384:307–316. doi:10.1042/BJ20040605 15361072 PMC1134114

[20] Tailford LE, Crost EH, Kavanaugh D, Juge N. 2015. Mucin glycan foraging in the human gut microbiome. Front Genet 6:81. doi:10.3389/fgene.2015.00081 25852737 PMC4365749

[21] Wlodarska M, Luo C, Kolde R, d’Hennezel E, Annand JW, Heim CE, Krastel P, Schmitt EK, Omar AS, Creasey EA, Garner AL, Mohammadi S, O’Connell DJ, Abubucker S, Arthur TD, Franzosa EA, Huttenhower C, Murphy LO, Haiser HJ, Vlamakis H, Porter JA, Xavier RJ. 2017. Indoleacrylic acid produced by commensal Peptostreptococcus species suppresses inflammation. Cell Host Microbe 22:25–37. doi:10.1016/j.chom.2017.06.007 28704649 PMC5672633

[22] Miller RS, Hoskins LC. 1981. Mucin degradation in human colon ecosystems: fecal population densities of mucin-degrading bacteria estimated by a most probable number method. Gastroenterology 81:759–765. doi:10.1016/0016-5085(81)90503-5 7262520

[23] Berkhout MD, Plugge CM, Belzer C. 2022. How microbial glycosyl hydrolase activity in the gut mucosa initiates microbial cross-feeding. Glycobiology 32:182–200. doi:10.1093/glycob/cwab105 34939101 PMC8966484

[24] Kostopoulos I, Aalvink S, Kovatcheva-Datchary P, Nijsse B, Bäckhed F, Knol J, de Vos WM, Belzer C. 2021. A continuous battle for host-derived glycans between a mucus specialist and a glycan generalist in vitro and in vivo. Front Microbiol 12:632454. doi:10.3389/fmicb.2021.632454 34248864 PMC8264420

[25] Salyers AA, Vercellotti JR, West SE, Wilkins TD. 1977. Fermentation of mucin and plant polysaccharides by strains of bacteroides from the human colon. Appl Environ Microbiol 33:319–322. doi:10.1128/aem.33.2.319-322.1977 848954 PMC170684

[26] Martens EC, Chiang HC, Gordon JI. 2008. Mucosal glycan foraging enhances fitness and transmission of a Saccharolytic human gut bacterial symbiont. Cell Host Microbe 4:447–457. doi:10.1016/j.chom.2008.09.007 18996345 PMC2605320

[27] Raba G, Luis AS. 2023. Mucin utilization by gut microbiota: recent advances on characterization of key enzymes. Essays Biochem 67:345–353. doi:10.1042/EBC20220121 36695502 PMC10154618

[28] Nakjang S, Ndeh DA, Wipat A, Bolam DN, Hirt RP. 2012. A novel extracellular metallopeptidase domain shared by animal host-associated mutualistic and pathogenic microbes. PLOS ONE 7:e30287. doi:10.1371/journal.pone.0030287 22299034 PMC3267712

[29] Shon DJ, Fernandez D, Riley NM, Ferracane MJ, Bertozzi CR. 2022. Structure-guided mutagenesis of a mucin-selective metalloprotease from Akkermansia muciniphila alters substrate preferences. J Biol Chem 298:101917. doi:10.1016/j.jbc.2022.101917 35405095 PMC9118916

[30] Luis AS, Jin C, Pereira GV, Glowacki RWP, Gugel SR, Singh S, Byrne DP, Pudlo NA, London JA, Baslé A, Reihill M, Oscarson S, Eyers PA, Czjzek M, Michel G, Barbeyron T, Yates EA, Hansson GC, Karlsson NG, Cartmell A, Martens EC. 2021. A single sulfatase is required to access colonic Mucin by a gut bacterium. Nature 598:332–337. doi:10.1038/s41586-021-03967-5 34616040 PMC9128668

[31] Png CW, Lindén SK, Gilshenan KS, Zoetendal EG, McSweeney CS, Sly LI, McGuckin MA, Florin THJ. 2010. Mucolytic bacteria with increased prevalence in IBD mucosa augment in vitro utilization of mucin by other bacteria. Am J Gastroenterol 105:2420–2428. doi:10.1038/ajg.2010.281 20648002

[32] Shuoker B, Pichler MJ, Jin C, Sakanaka H, Wu H, Gascueña AM, Liu J, Nielsen TS, Holgersson J, Nordberg Karlsson E, Juge N, Meier S, Morth JP, Karlsson NG, Abou Hachem M. 2023. Sialidases and fucosidases of Akkermansia muciniphila are crucial for growth on mucin and nutrient sharing with mucus-associated gut bacteria. Nat Commun 14:1833. doi:10.1038/s41467-023-37533-6 37005422 PMC10067855

[33] Glover JS, Ticer TD, Engevik MA. 2022. Characterizing the mucin-degrading capacity of the human gut microbiota. Sci Rep 12:8456. doi:10.1038/s41598-022-11819-z 35589783 PMC9120202

[34] Kansal S, Catto-Smith AG, Boniface K, Thomas S, Cameron DJ, Oliver M, Alex G, Kirkwood CD, Wagner J. 2019. Variation of gut mucosal microbiome with anti-Saccharomyces cerevisiae antibody status in pediatric Crohn disease. J Pediatr Gastroenterol Nutr 69:696–703. doi:10.1097/MPG.0000000000002461 31764438

[35] Raygoza Garay JA, Turpin W, Lee S-H, Smith MI, Goethel A, Griffiths AM, Moayyedi P, Espin-Garcia O, Abreu M, Aumais GL, et al.. 2023. Gut microbiome composition is associated with future onset of Crohn’s disease in healthy first-degree relatives. Gastroenterology 165:670–681. doi:10.1053/j.gastro.2023.05.032 37263307

[36] Salyers AA, West SE, Vercellotti JR, Wilkins TD. 1977. Fermentation of mucins and plant Polysaccharides by anaerobic bacteria from the human colon. Appl Environ Microbiol 34:529–533. doi:10.1128/aem.34.5.529-533.1977 563214 PMC242695

[37] Hoskins LC, Agustines M, McKee WB, Boulding ET, Kriaris M, Niedermeyer G. 1985. Mucin degradation in human colon ecosystems. isolation and properties of fecal strains that degrade ABH blood group antigens and oligosaccharides from mucin glycoproteins. J Clin Invest 75:944–953. doi:10.1172/JCI111795 3920248 PMC423632

[38] Larson G, Falk P, Hoskins LC. 1988. Degradation of human intestinal glycosphingolipids by extracellular glycosidases from mucin-degrading bacteria of the human fecal flora. J Biol Chem 263:10790–10798. 3392043

[39] Lloyd-Price J, Arze C, Ananthakrishnan AN, Schirmer M, Avila-Pacheco J, Poon TW, Andrews E, Ajami NJ, Bonham KS, Brislawn CJ, et al.. 2019. Multi-omics of the gut microbial ecosystem in inflammatory bowel diseases. Nature 569:655–662. doi:10.1038/s41586-019-1237-9 31142855 PMC6650278

[40] Davey LE, Malkus PN, Villa M, Dolat L, Holmes ZC, Letourneau J, Ansaldo E, David LA, Barton GM, Valdivia RH. 2023. A genetic system for Akkermansia muciniphila reveals a role for mucin foraging in gut colonization and host sterol biosynthesis gene expression. Nat Microbiol 8:1450–1467. doi:10.1038/s41564-023-01407-w 37337046 PMC11741908

[41] Pudlo NA, Urs K, Crawford R, Pirani A, Atherly T, Jimenez R, Terrapon N, Henrissat B, Peterson D, Ziemer C, Snitkin E, Martens EC. 2022. Phenotypic and genomic diversification in complex carbohydrate-degrading human gut bacteria. mSystems 7:e0094721. doi:10.1128/msystems.00947-21 35166563 PMC8845570

[42] Pudlo NA, Urs K, Kumar SS, German JB, Mills DA, Martens EC. 2015. Symbiotic human gut bacteria with variable metabolic priorities for host mucosal glycans. mBio 6:e01282–15. doi:10.1128/mBio.01282-15 26556271 PMC4659458

[43] Drula E, Garron M-L, Dogan S, Lombard V, Henrissat B, Terrapon N. 2022. The carbohydrate-active enzyme database: functions and literature. Nucleic Acids Res 50:D571–D577. doi:10.1093/nar/gkab1045 34850161 PMC8728194

[44] Cheng K, Zhou Y, Neelamegham S. 2017. DrawGlycan-SNFG: a robust tool to render glycans and glycopeptides with fragmentation information. Glycobiology 27:200–205. doi:10.1093/glycob/cww115 28177454 PMC6410959

[45] Karlsson NG, Jin C, Rojas-Macias MA, Adamczyk B. 2017. Next generation O-linked glycomics. Trends Glycosci. Glycotechnol 29:E35–E46. doi:10.4052/tigg.1602.1E

[46] 2009. Essentials of Glycobiology. Cold Spring Harbor Laboratory Press, Cold Spring Harbor (NY). 20301239

[47] Ostrowski MP, La Rosa SL, Kunath BJ, Robertson A, Pereira G, Hagen LH, Varghese NJ, Qiu L, Yao T, Flint G, et al.. 2022. Mechanistic insights into consumption of the food additive xanthan gum by the human gut microbiota. Nat Microbiol 7:556–569. doi:10.1038/s41564-022-01093-0 35365790 PMC11537241

[48] Hehemann J-H, Kelly AG, Pudlo NA, Martens EC, Boraston AB. 2012. Bacteria of the human gut microbiome catabolize red seaweed glycans with carbohydrate-active enzyme updates from extrinsic Microbes. Proc Natl Acad Sci U S A 109:19786–19791. doi:10.1073/pnas.1211002109 23150581 PMC3511707

[49] Koropatkin NM, Martens EC, Gordon JI, Smith TJ. 2008. Starch catabolism by a prominent human gut symbiont is directed by the recognition of amylose helices. Structure 16:1105–1115. doi:10.1016/j.str.2008.03.017 18611383 PMC2563962

[50] Crouch LI, Liberato MV, Urbanowicz PA, Baslé A, Lamb CA, Stewart CJ, Cooke K, Doona M, Needham S, Brady RR, Berrington JE, Madunic K, Wuhrer M, Chater P, Pearson JP, Glowacki R, Martens EC, Zhang F, Linhardt RJ, Spencer DIR, Bolam DN. 2020. Prominent members of the human gut microbiota express endo-acting O-glycanases to initiate mucin breakdown. Nat Commun 11:4017. doi:10.1038/s41467-020-17847-5 32782292 PMC7419316

[51] Ze X, Le Mougen F, Duncan SH, Louis P, Flint HJ. 2013. Some are more equal than others. Gut Microbes 4:236–240. doi:10.4161/gmic.23998 23549436 PMC3669169

[52] Ze X, Duncan SH, Louis P, Flint HJ. 2012. Ruminococcus bromii is a keystone species for the degradation of resistant starch in the human colon. ISME J 6:1535–1543. doi:10.1038/ismej.2012.4 22343308 PMC3400402

[53] Miron J, Duncan SH, Stewart CS. 1994. Interactions between rumen bacterial strains during the degradation and utilization of the monosaccharides of barley straw cell-walls. J Appl Bacteriol 76:282–287. doi:10.1111/j.1365-2672.1994.tb01629.x 8157547

[54] Martens EC, Roth R, Heuser JE, Gordon JI. 2009. Coordinate regulation of glycan degradation and polysaccharide capsule biosynthesis by a prominent human gut symbiont. J Biol Chem 284:18445–18457. doi:10.1074/jbc.M109.008094 19403529 PMC2709373

[55] Bjursell MK, Martens EC, Gordon JI. 2006. Functional genomic and metabolic studies of the adaptations of a prominent adult human gut symbiont, bacteroides thetaiotaomicron, to the suckling period*. J Biol Chem 281:36269–36279. doi:10.1074/jbc.M606509200 16968696

[56] Shimada Y, Watanabe Y, Wakinaka T, Funeno Y, Kubota M, Chaiwangsri T, Kurihara S, Yamamoto K, Katayama T, Ashida H. 2015. α-N-acetylglucosaminidase from Bifidobacterium bifidum specifically hydrolyzes α-linked N-acetylglucosamine at nonreducing terminus of O-glycan on gastric mucin. Appl Microbiol Biotechnol 99:3941–3948. doi:10.1007/s00253-014-6201-x 25381911

[57] Wada J, Ando T, Kiyohara M, Ashida H, Kitaoka M, Yamaguchi M, Kumagai H, Katayama T, Yamamoto K. 2008. Bifidobacterium bifidum lacto-N-biosidase, a critical enzyme for the degradation of human milk oligosaccharides with a type 1 structure. Appl Environ Microbiol 74:3996–4004. doi:10.1128/AEM.00149-08 18469123 PMC2446520

[58] Takada H, Katoh T, Sakanaka M, Odamaki T, Katayama T. 2023. GH20 and GH84 β-N-acetylglucosaminidases with different linkage specificities underpin mucin O-glycan breakdown capability of Bifidobacterium bifidum. J Biol Chem 299:104781. doi:10.1016/j.jbc.2023.104781 37146969 PMC10245121

[59] Falk P, Hoskins LC, Larson G. 1991. Enhancing effects of bile salts on the degradation of glycosphingolipids by glycosidases from bacteria of the human fecal flora. Biochim Biophys Acta BBA - Lipids Lipid Metab 1084:139–148. doi:10.1016/0005-2760(91)90212-Z 1854798

[60] Seregin SS, Golovchenko N, Schaf B, Chen J, Pudlo NA, Mitchell J, Baxter NT, Zhao L, Schloss PD, Martens EC, Eaton KA, Chen GY. 2017. NLRP6 protects Il10−/− mice from colitis by limiting colonization of Akkermansia muciniphila. Cell Rep 19:733–745. doi:10.1016/j.celrep.2017.03.080 28445725 PMC5528001

[61] Hickey CA, Kuhn KA, Donermeyer DL, Porter NT, Jin C, Cameron EA, Jung H, Kaiko GE, Wegorzewska M, Malvin NP, Glowacki RWP, Hansson GC, Allen PM, Martens EC, Stappenbeck TS. 2015. Colitogenic bacteroides thetaiotaomicron antigens access host immune cells in a sulfatase-dependent manner via outer membrane vesicles. Cell Host Microbe 17:672–680. doi:10.1016/j.chom.2015.04.002 25974305 PMC4432250

[62] Manzi AE, Norgard-Sumnicht K, Argade S, Marth JD, van Halbeek H, Varki A. 2000. Exploring the glycan repertoire of genetically modified mice by isolation and profiling of the major glycan classes and nano-NMR analysis of glycan mixtures. Glycobiology 10:669–689. doi:10.1093/glycob/10.7.669 10910972

[63] Carlstedt I, Herrmann A, Karlsson H, Sheehan J, Fransson LA, Hansson GC. 1993. Characterization of two different glycosylated domains from the insoluble mucin complex of rat small intestine. J Biol Chem 268:18771–18781. doi:10.1016/S0021-9258(17)46696-8 8360170

[64] 1975. Anaerobe laboratory manual. The Laboratory, Blacksburg, Va.

[65] Noach I, Ficko-Blean E, Pluvinage B, Stuart C, Jenkins ML, Brochu D, Buenbrazo N, Wakarchuk W, Burke JE, Gilbert M, Boraston AB. 2017. Recognition of protein-linked glycans as a determinant of peptidase activity. Proc Natl Acad Sci U S A 114:E679–E688. doi:10.1073/pnas.1615141114 28096352 PMC5293097

[66] Porter NT, Canales P, Peterson DA, Martens EC. 2017. A subset of polysaccharide capsules in the human symbiont Bacteroides thetaiotaomicron promote increased competitive fitness in the mouse gut. Cell Host Microbe 22:494–506. doi:10.1016/j.chom.2017.08.020 28966055 PMC5830307

[67] Liu W, Xie Y, Ma J, Luo X, Nie P, Zuo Z, Lahrmann U, Zhao Q, Zheng Y, Zhao Y, Xue Y, Ren J. 2015. IBS: an illustrator for the presentation and visualization of biological sequences. Bioinformatics 31:3359–3361. doi:10.1093/bioinformatics/btv362 26069263 PMC4595897

[68] Almagro Armenteros JJ, Tsirigos KD, Sønderby CK, Petersen TN, Winther O, Brunak S, von Heijne G, Nielsen H. 2019. SignalP 5.0 improves signal peptide predictions using deep neural networks. Nat Biotechnol 37:420–423. doi:10.1038/s41587-019-0036-z 30778233

[69] Nagao-Kitamoto H, Leslie JL, Kitamoto S, Jin C, Thomsson KA, Gillilland MG 3rd, Kuffa P, Goto Y, Jenq RR, Ishii C, Hirayama A, Seekatz AM, Martens EC, Eaton KA, Kao JY, Fukuda S, Higgins PDR, Karlsson NG, Young VB, Kamada N. 2020. Interleukin-22-mediated host glycosylation prevents Clostridioides difficile infection by modulating the metabolic activity of the gut microbiota. Nat Med 26:608–617. doi:10.1038/s41591-020-0764-0 32066975 PMC7160049

