# Supplementary material for: Ruminococcus torques is a keystone degrader of intestinal mucin glycoprotein, releasing oligosaccharides used by Bacteroides thetaiotaomicron
Source: mBio. 2024 Jul 8;15(8):e00039-24. doi: 10.1128/mbio.00039-24 (PMC11323728; doi:10.1128/mbio.00039-24)
Supplement: Supplemental Figures — Figures S1 to S13. [file mbio.00039-24-s0001.pdf]

Figure S1

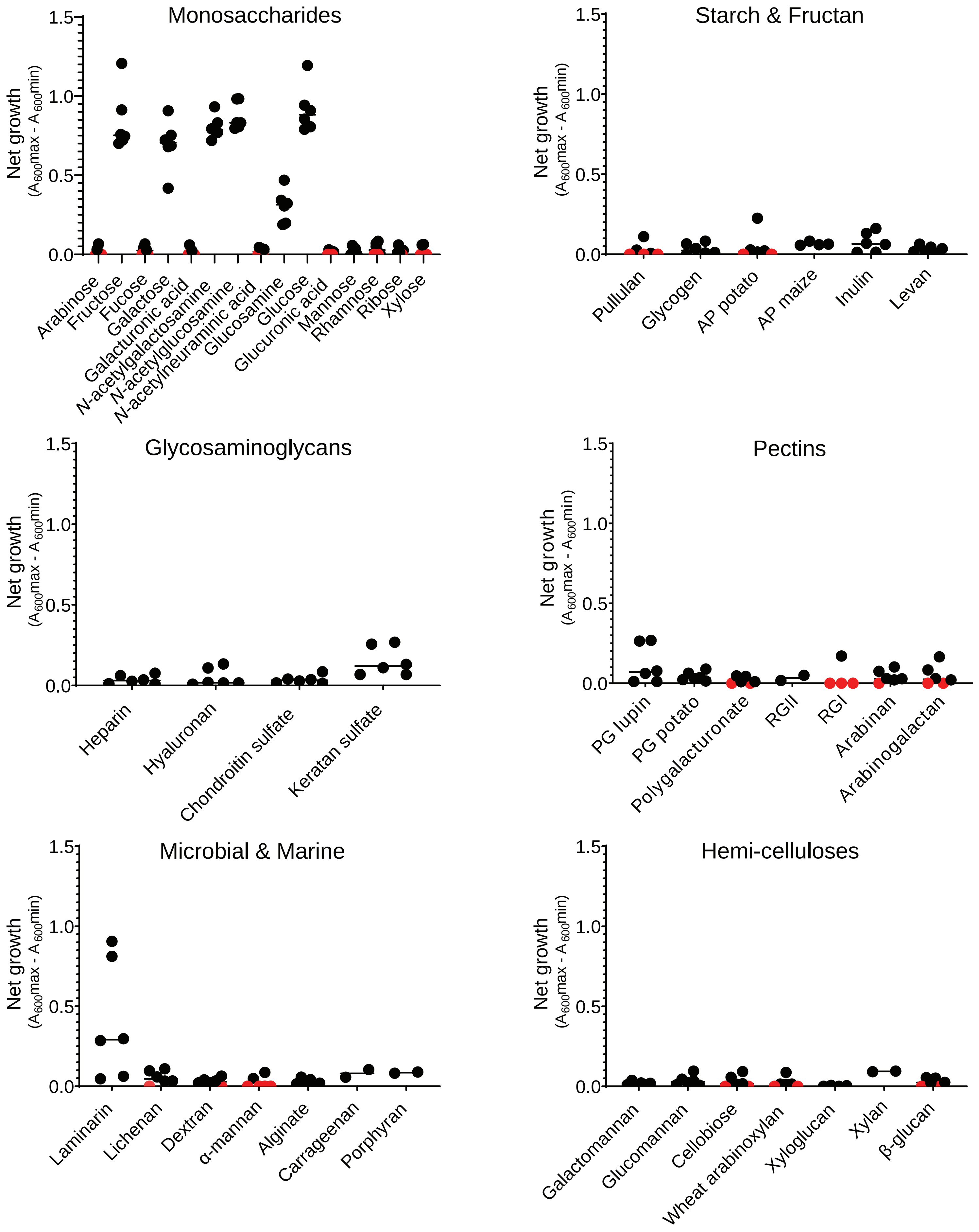
Monosaccharides
Starch & Fructan
Glycosaminoglycans
Pectins
Microbial & Marine
Hemi-celluloses
Net growth
(A600max - A600min)
1.5
1.0
0.5
0.0
Arabinose
Fructose
Fucose
Galactose
Galacturonic acid
N-acetylgalactosamine
N-acetylglucosamine
N-acetylneuraminic acid
Glucosamine
Glucose
Glucuronic acid
Mannose
Rhamnose
Ribose
Xylose
Pullulan
Glycogen
AP potato
AP maize
Inulin
Levan
Heparin
Hyaluronan
Chondroitin sulfate
Keratan sulfate
PG lupin
PG potato
Polygalacturonate
RGII
RGI
Arabinan
Arabinogalactan
Laminarin
Lichenan
Dextran
α-mannan
Alginate
Carrageenan
Porphyran
Galactomannan
Glucomannan
Cellobiose
Wheat arabinoxylan
Xyloglucan
Xylan
β-glucan

Supplemental Figure S1. *R. torques* growth on various carbohydrates. *R. torques* was grown on 46 substrates, each as the main carbohydrate source in YCFA medium. Growth was measured by absorbance at 600 nm for ~92 hours. Growth curves were analyzed to identify minimum and maximum absorbance values for each curve (excluding values representing artifacts due to brief disturbances of the plates or baseline noise), and the net growth (max absorbance – min absorbance) was calculated. For growths in which the absorbance consistently decreased throughout the growth curve with no apparent increase in absorbance, net growth was reported as zero, and these points are displayed in red. AP = amylopectin, PG = pectic galactan, RG = rhamnogalacturonan (type I or type II specified).

Figure S2

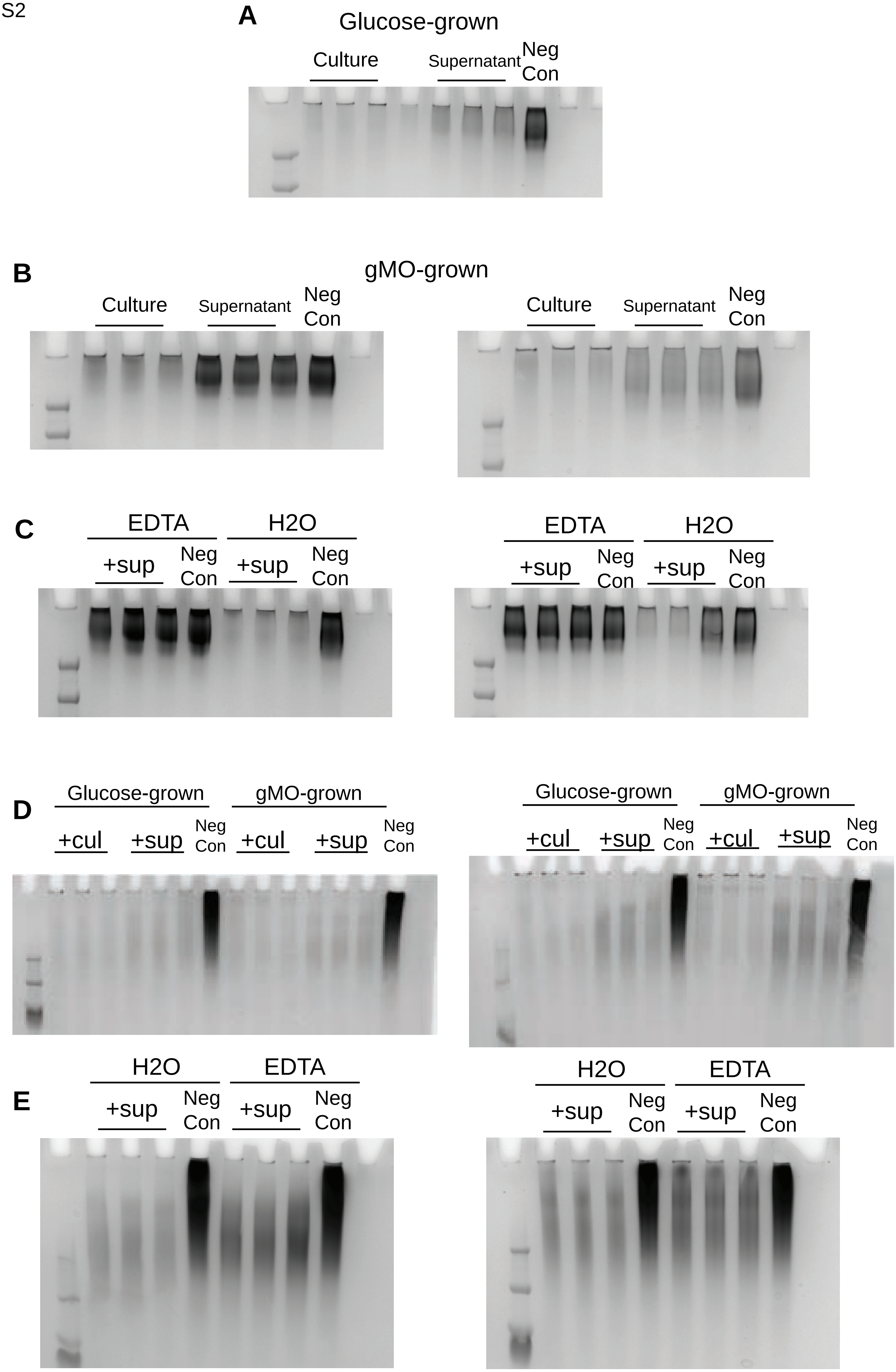

A
Glucose-grown
Culture
Supernatant
Neg Con
B
gMO-grown
Culture
Supernatant
Neg Con
Culture
Supernatant
Neg Con
C
EDTA
H2O
+sup
Neg Con
+sup
Neg Con
EDTA
H2O
+sup
Neg Con
+sup
Neg Con
D
Glucose-grown
gMO-grown
+cul
+sup
Neg Con
+cul
+sup
Neg Con
Glucose-grown
gMO-grown
+cul
+sup
Neg Con
+cul
+sup
Neg Con
E
H2O
EDTA
+sup
Neg Con
+sup
Neg Con
H2O
EDTA
+sup
Neg Con
+sup
Neg Con

Supplemental Figure S2. Periodic acid-Schiff-stained gels to visualize degradation of mucin glycoproteins by *R. torques.* Culture (*i.e.*, cells and supernatants) or cell-free supernatant samples were collected from *R. torques* after overnight growth on glucose or gMO. Samples were incubated with cMUC2 or PGM anaerobically for 2 days and electrophoresed through 4-12% Bis-tris gels and stained with periodic acid-Schiff stain to visualize mucins. **A-C**, Degradation of cMUC2 (2.5 mg/ml final concentration) by glucose-grown samples (**A**), gMO-grown samples (**B**), or 10mM EDTA treated samples (**C**). **D-E**, degradation of PGM (10 mg/ml final concentration) by glucose or gMO-grown samples (**D**) or 10 mM EDTA treated samples (**E**).

Figure S3

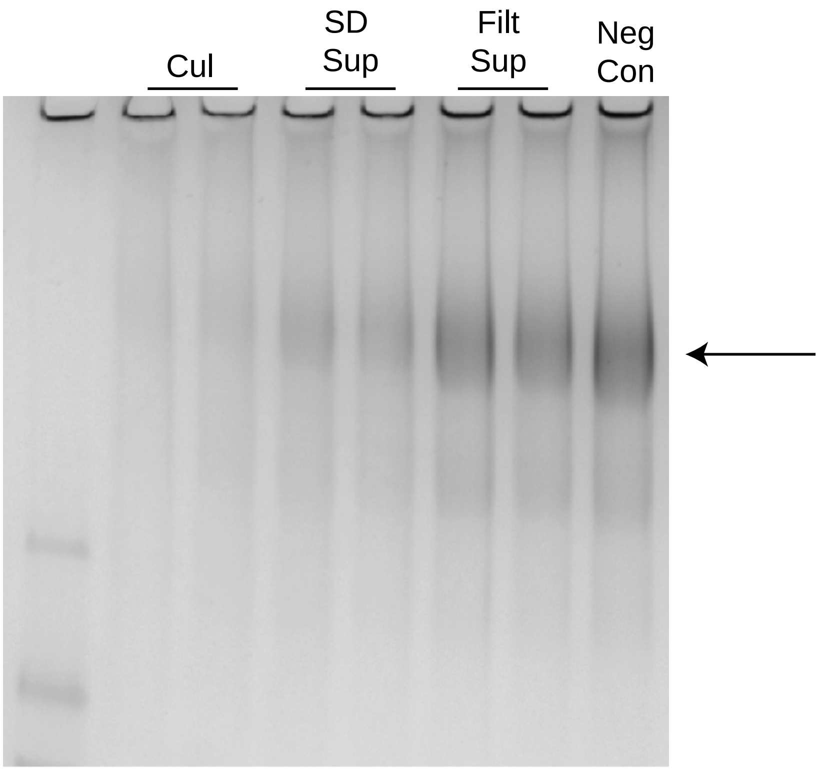

Supplemental Figure S3. Degradation of cMUC2 by *R. torques* supernatants after centrifugation or filtering. *R. torques* was grown overnight in YCFA with glucose and samples were either centrifuged or filtered (0.22 um PVDF filters) to collect supernatants. Supernatants were then incubated with cMUC2 anaerobically for 2 days. Samples were analyzed by electrophoresis through a 4-12% Tris-Glycine gel and stained with periodic acid-Schiff stain to visualize mucin (major band indicated by arrow). Cul = whole culture (cells + supernatant), SD sup = spun-down (centrifuged) supernatant, Filt sup = filtered supernatant, Neg con = negative control (YCFA with glucose added in lieu of *R. torques* cultures or supernatant).

Figure S4

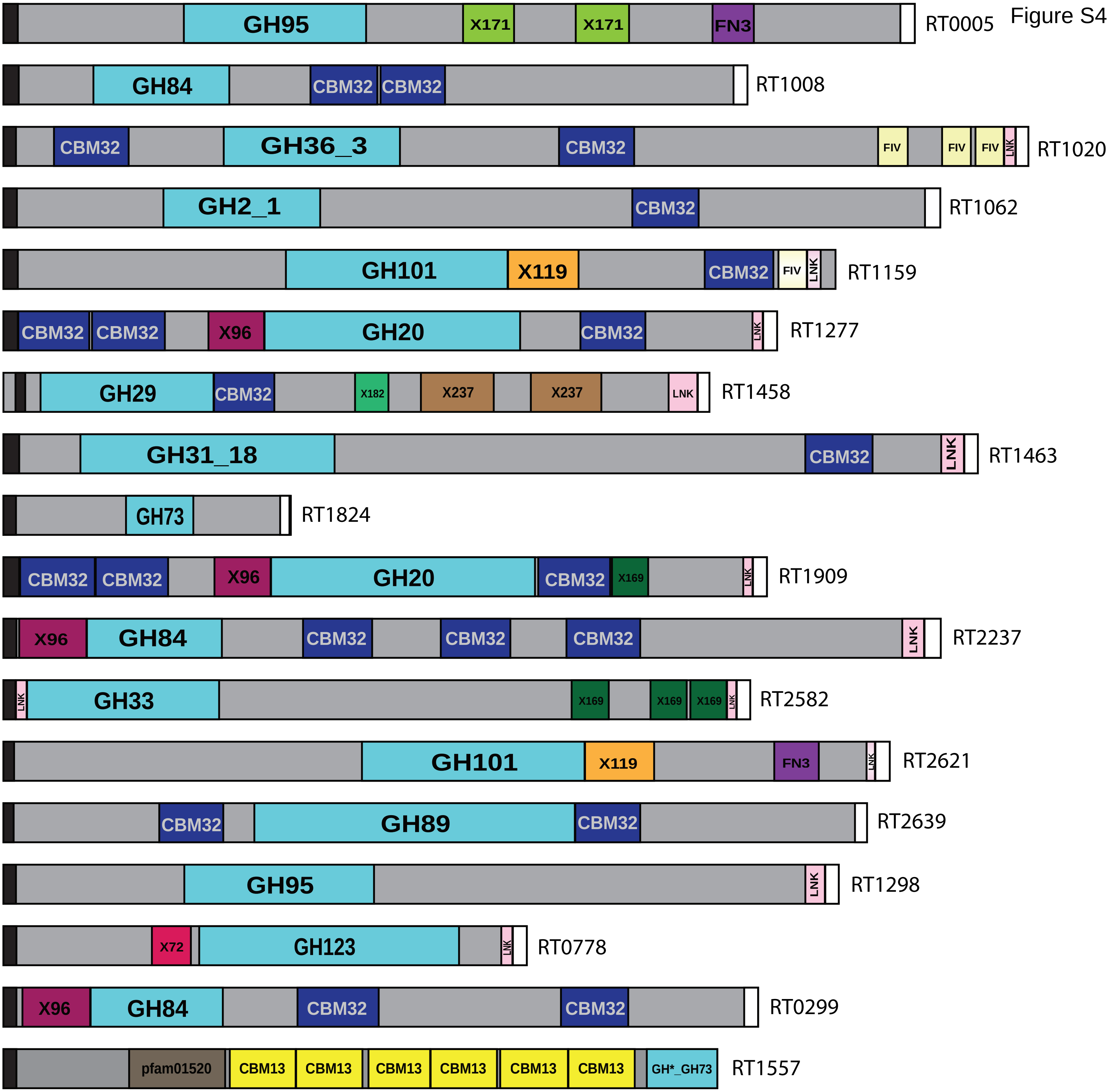
GH95
X171
X171
FN3
RT0005
GH84
CBM32
CBM32
RT1008
CBM32
GH36_3
CBM32
FIV
FIV
FIV
LNK
RT1020
GH2_1
CBM32
RT1062
GH101
X119
CBM32
FIV
LNK
RT1159
CBM32
CBM32
X96
GH20
CBM32
LNK
RT1277
GH29
CBM32
X182
X237
X237
LNK
RT1458
GH31_18
CBM32
LNK
RT1463
GH73
RT1824
CBM32
CBM32
X96
GH20
CBM32
X169
LNK
RT1909
X96
GH84
CBM32
CBM32
CBM32
LNK
RT2237
LNK
GH33
X169
X169
X169
LNK
RT2582
GH101
X119
FN3
LNK
RT2621
CBM32
GH89
CBM32
RT2639
GH95
LNK
RT1298
X72
GH123
LNK
RT0778
X96
GH84
CBM32
CBM32
RT0299
pfam01520
CBM13
CBM13
CBM13
CBM13
CBM13
CBM13
GH*_GH73
RT1557

Supplemental Figure S4. Predicted domain architecture of highly expressed and transcribed CAZymes. Domain architecture predictions were generated using CAZy. Figure made with Illustrator for Biological Sequences[70]. Black = signal peptide; white = transmembrane domain; teal = CAZyme domains; blue = CBM domains. GH*_GH73 denotes a distant relationship of that domain to the GH73 family.

Figure S5

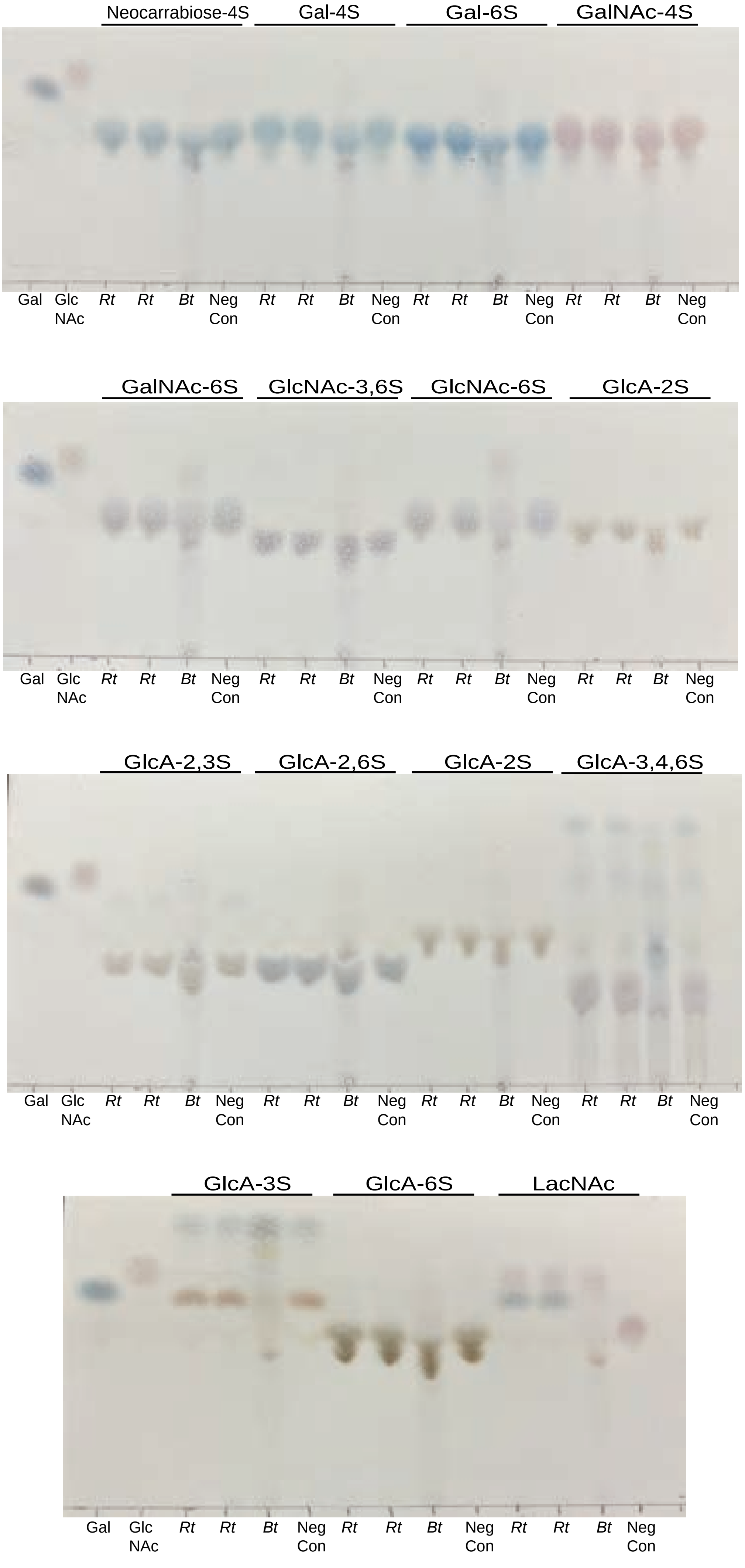
Neocarrabiose-4S
Gal-4S
Gal-6S
GalNAc-4S
Gal Glc NAc Rt Rt Bt Neg Con Rt Rt Bt Neg Con Rt Rt Bt Neg Con Rt Rt Bt Neg Con
GalNAc-6S
GlcNAc-3,6S
GlcNAc-6S
GlcA-2S
Gal Glc NAc Rt Rt Bt Neg Con Rt Rt Bt Neg Con Rt Rt Bt Neg Con Rt Rt Bt Neg Con
GlcA-2,3S
GlcA-2,6S
GlcA-2S
GlcA-3,4,6S
Gal Glc NAc Rt Rt Bt Neg Con Rt Rt Bt Neg Con Rt Rt Bt Neg Con Rt Rt Bt Neg Con
GlcA-3S
GlcA-6S
LacNAc
Gal Glc NAc Rt Rt Bt Neg Con Rt Rt Bt Neg Con Rt Rt Bt Neg Con

Supplemental Figure S5. *R. torques* fails to degrade sulfated sugars. Proteins were ammonium sulfate precipitated from the supernatant of *R. torques* overnight cultures after growth in YCFA with glucose and incubated with respective sugars (5mM final concentration, except for GlcA-6-O-sulfate which was 20 mM final concentration). Reactions were analyzed by thin layer chromatography using diphenylamine-aniline-phosphoric acid developer. *Rt* indicates samples where *R. torques* precipitated proteins were added, *Bt* indicates samples where sonicated *B. thetaiotaomicron* cultures exposed to keratan sulfate was added, and Neg Con indicates negative control samples that contained buffer (10 mM MES and 5 mM $CaCl_2$). Gal = galactose; GalNAc = *N*-acetylgalactosamine; GlcNAc = *N*-acetylglucosamine; GalA = galactosamine; GlcA = glucosamine.

Figure S6

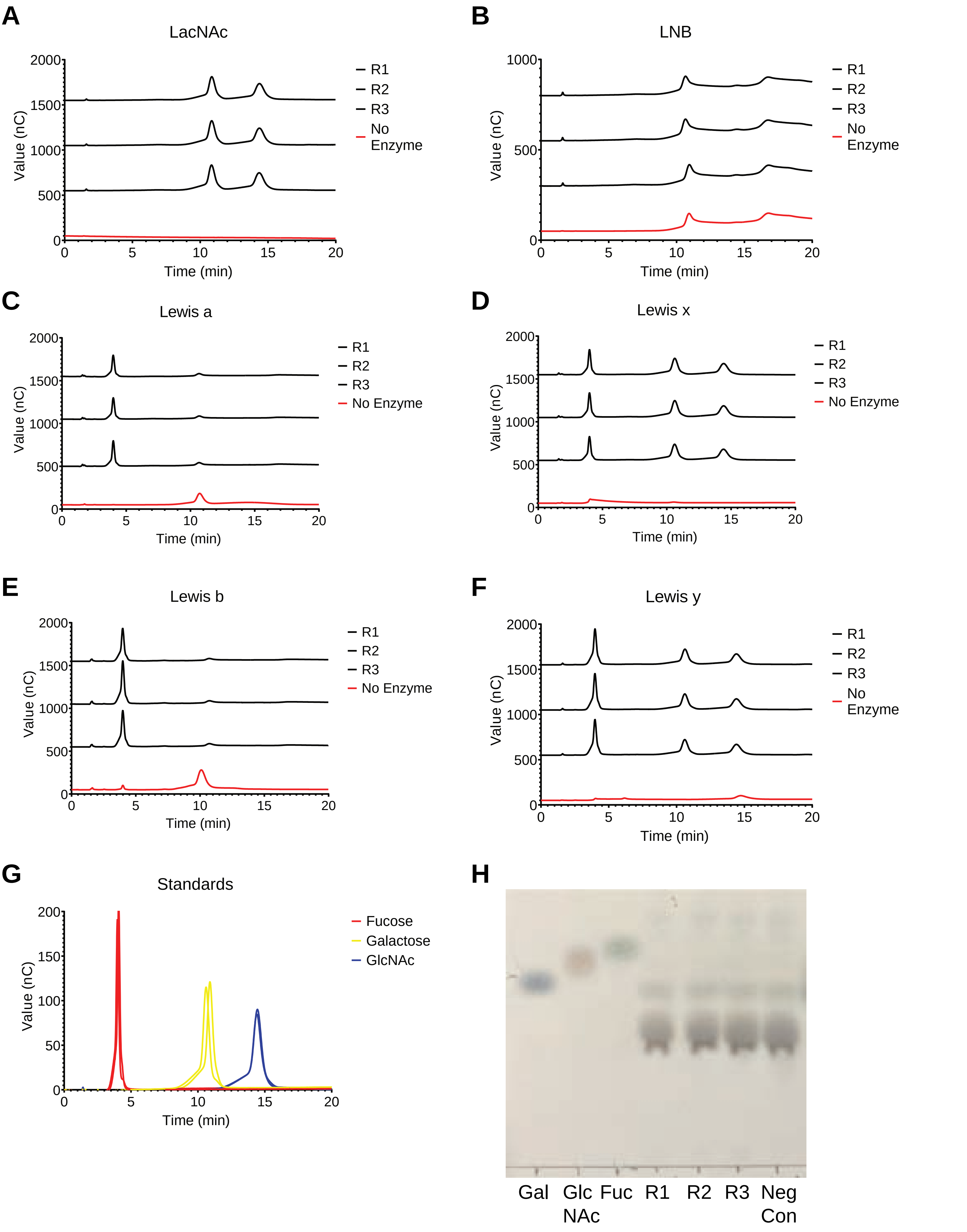
A
LacNAc
Value (nC)
Time (min)
R1
R2
R3
No Enzyme
B
LNB
C
Lewis a
D
Lewis x
E
Lewis b
F
Lewis y
G
Standards
Fucose
Galactose
GlcNAc
H
Gal
Glc NAc
Fuc
R1
R2
R3
Neg Con

Supplemental Figure S6. HPAEC-PAD analysis of *R. torques* ammonium sulfate precipitated protein activity on model mucin sugars. A-G, Chromatograms collected by HPAEC-PAD analysis of *R. torques* supernatant precipitated protein reactions with model glycans *N*-acetyllactosamine (LacNAc, **A**), and lacto-*N*-biose (LNB, **B**), Lewis a (**C**), x (**D**), b (**E**), y (**F**) compared to single monosaccharide standards (**G**). R1-R3 = replicates reactions with supernatant precipitates from 3 *R. torques* cultures. No enzyme control is shown in red, where PBS was added instead of *R. torques* precipitates. **H**, Thin layer chromatogram of reaction products of activity assay on lacto-*N*-biose (LNB) from A. R1-R3=replicate reactions with *R. torques* supernatant precipitates; Neg Con=no enzyme control reaction with PBS.

Figure S7

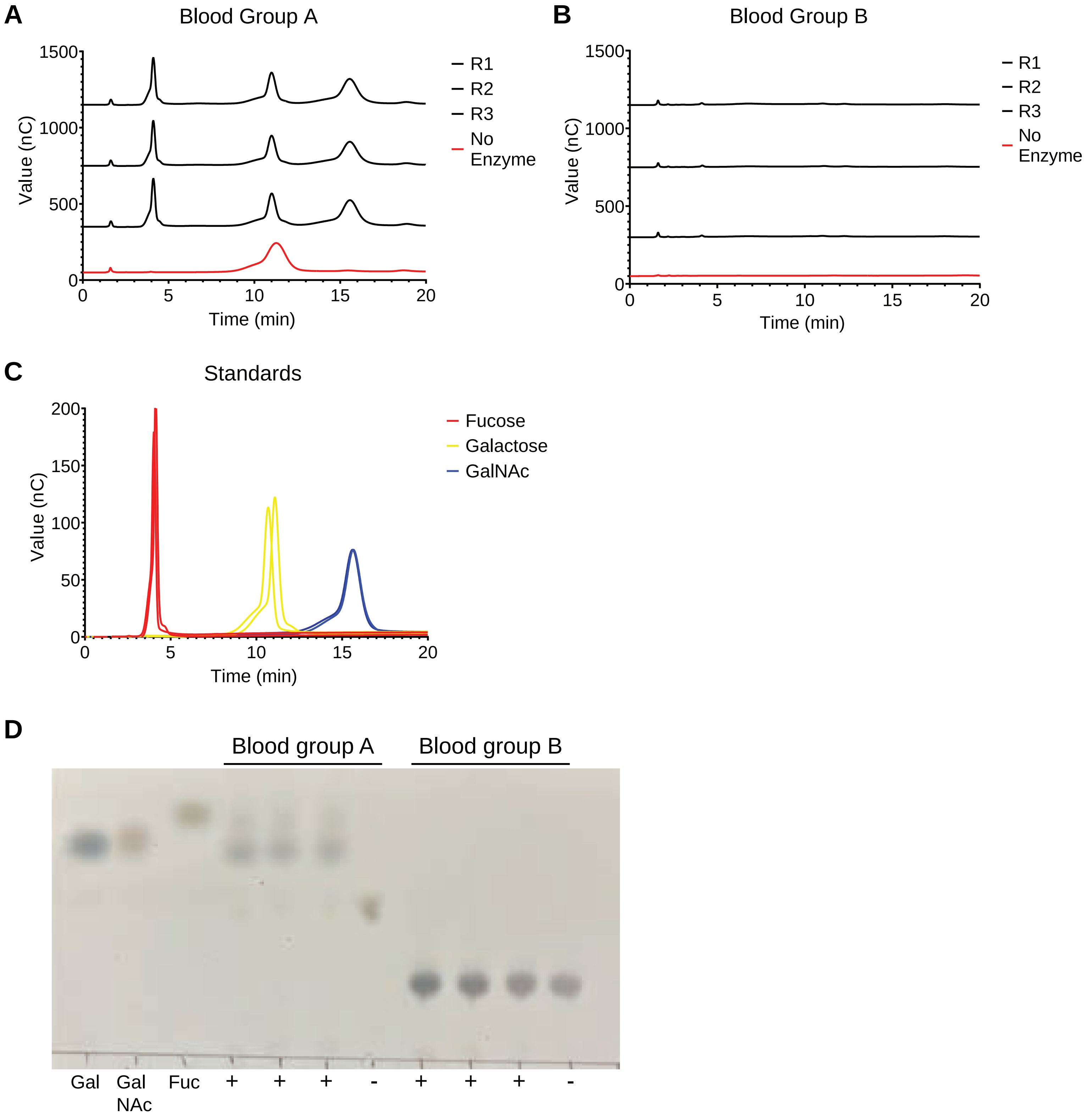
A
Blood Group A
1500
1000
500
0
Value (nC)
0
5
10
15
20
Time (min)
R1
R2
R3
No Enzyme
B
Blood Group B
1500
1000
500
0
Value (nC)
0
5
10
15
20
Time (min)
R1
R2
R3
No Enzyme
C
Standards
200
150
100
50
0
Value (nC)
0
5
10
15
20
Time (min)
Fucose
Galactose
GalNAc
D
Blood group A
Blood group B
Gal
Gal NAc
Fuc
+
+
+
-
+
+
+
-

Supplemental Figure S7. HPAEC-PAD analysis of *R. torques* ammonium sulfate precipitated protein activity on blood groups A and B. A-C, Chromatograms collected by HPAEC-PAD analysis of *R. torques* supernatant precipitated protein reactions with model glycans blood groups A (**A**) and B (**B**) compared to single monosaccharide standards (**C**). R1-R3 = replicates reactions with supernatant precipitates from 3 *R. torques* cultures. No enzyme control is shown in red, where PBS was added instead of *R. torques* precipitates. **D**, Thin layer chromatogram of blood group A and blood group B reactions from A. Gal=galactose, GlcNAc=*N*-acetylglucosamine, Fuc=fucose, R1-R3=replicate reactions with *R. torques* supernatant precipitates, Neg Con=no enzyme control reaction with PBS.

Figure S8

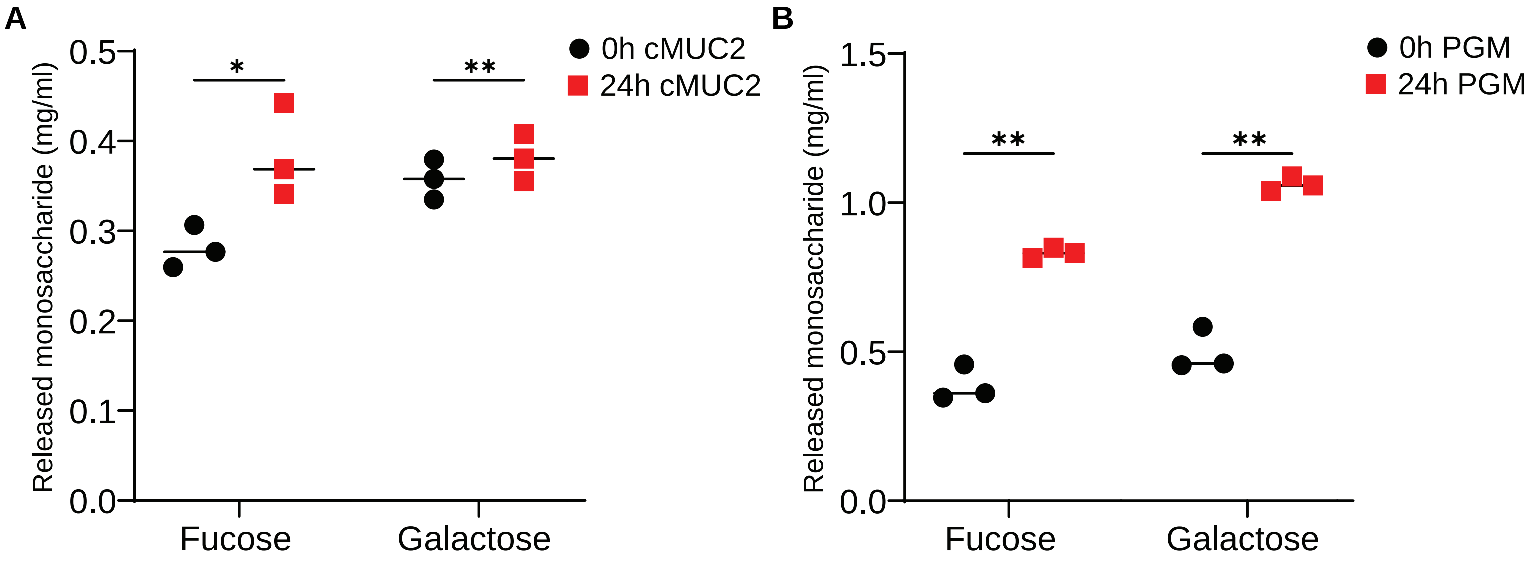
A
0h cMUC2
24h cMUC2
Released monosaccharide (mg/ml)
0.0
0.1
0.2
0.3
0.4
0.5
*
**
Fucose
Galactose
B
0h PGM
24h PGM
Released monosaccharide (mg/ml)
0.0
0.5
1.0
1.5
**
**
Fucose
Galactose

Supplemental Figure S8. Fucose and galactose released from cMUC2 and PGM after digestion by supernatants from *R. torques* grown on gMO. A-B, Concentration of fucose or galactose released after 24-hour incubation of *R. torques* supernatants from cultures grown on gMO with cMUC2 (2.5 mg/ml final; **A**) or PGM (10 mg/ml final; **B**). Statistics were analyzed with paired, two-tailed t tests comparing release of each monosaccharide between substrates in each panel. *p<0.05, **p<0.01.

Figure S9

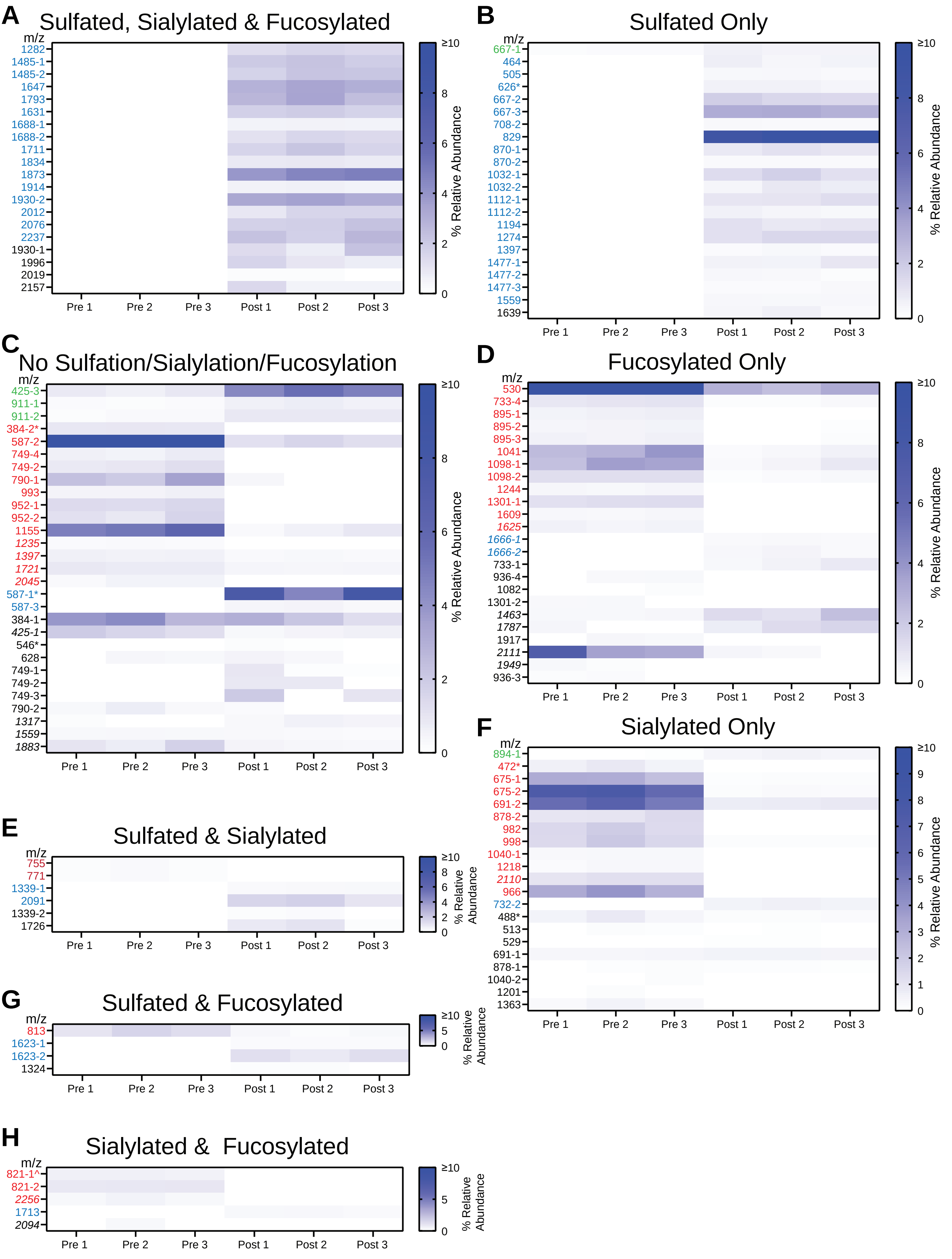
A
Sulfated, Sialylated & Fucosylated
m/z
1282
1485-1
1485-2
1647
1793
1631
1688-1
1688-2
1711
1834
1873
1914
1930-2
2012
2076
2237
1930-1
1996
2019
2157
Pre 1
Pre 2
Pre 3
Post 1
Post 2
Post 3
≥10
8
6
4
2
0
% Relative Abundance
B
Sulfated Only
m/z
667-1
464
505
626*
667-2
667-3
708-2
829
870-1
870-2
1032-1
1032-2
1112-1
1112-2
1194
1274
1397
1477-1
1477-2
1477-3
1559
1639
Pre 1
Pre 2
Pre 3
Post 1
Post 2
Post 3
≥10
8
6
4
2
0
% Relative Abundance
C
No Sulfation/Sialylation/Fucosylation
m/z
425-3
911-1
911-2
384-2*
587-2
749-4
749-2
790-1
993
952-1
952-2
1155
1235
1397
1721
2045
587-1*
587-3
384-1
425-1
546*
628
749-1
749-2
749-3
790-2
1317
1559
1883
Pre 1
Pre 2
Pre 3
Post 1
Post 2
Post 3
≥10
8
6
4
2
0
% Relative Abundance
D
Fucosylated Only
m/z
530
733-4
895-1
895-2
895-3
1041
1098-1
1098-2
1244
1301-1
1609
1625
1666-1
1666-2
733-1
936-4
1082
1301-2
1463
1787
1917
2111
1949
936-3
Pre 1
Pre 2
Pre 3
Post 1
Post 2
Post 3
≥10
8
6
4
2
0
% Relative Abundance
E
Sulfated & Sialylated
m/z
755
771
1339-1
2091
1339-2
1726
Pre 1
Pre 2
Pre 3
Post 1
Post 2
Post 3
≥10
8
6
4
2
0
% Relative Abundance
F
Sialylated Only
m/z
894-1
472*
675-1
675-2
691-2
878-2
982
998
1040-1
1218
2110
966
732-2
488*
513
529
691-1
878-1
1040-2
1201
1363
Pre 1
Pre 2
Pre 3
Post 1
Post 2
Post 3
≥10
9
8
7
6
5
4
3
2
1
0
% Relative Abundance
G
Sulfated & Fucosylated
m/z
813
1623-1
1623-2
1324
Pre 1
Pre 2
Pre 3
Post 1
Post 2
Post 3
≥10
5
0
% Relative Abundance
H
Sialylated & Fucosylated
m/z
821-1^
821-2
2256
1713
2094
Pre 1
Pre 2
Pre 3
Post 1
Post 2
Post 3
≥10
5
0
% Relative Abundance

Supplemental Figure S9. LC-MS/MS glycans identified before and after growth of *R. torques* on cMUC2. A-H, Glycans detected were grouped based on structural features: sulfated, sialylated and fucosylated (**A**), sulfated only (**B**), no sulfation, sialylation, fucosylation (**C**), fucosylated only (**D**), sulfated and sialylated (**E**), sialylated only (**F**), sulfated and fucosylated (**G**), and sialylated and fucosylated (**H**). Heat maps indicate percent relative abundance of each glycan pre- or post-growth of *R. torques.* M/z values in green represent glycans detected in at least one pre-growth sample that significantly increase in abundance post-growth; glycans in red decreased in abundance post-growth compared to pre-growth, glycans in blue were undetected pre-growth but were detected post-growth; and glycans in black were not significantly different in abundance pre- or post-growth. Statistics were analyzed using paired, two-tailed t-tests between the three pre- and three post-growth samples for each glycan. * = peeling reaction product, ^ = fuc-ol terminating glycan, italicized m/z values = *N*-glycan.

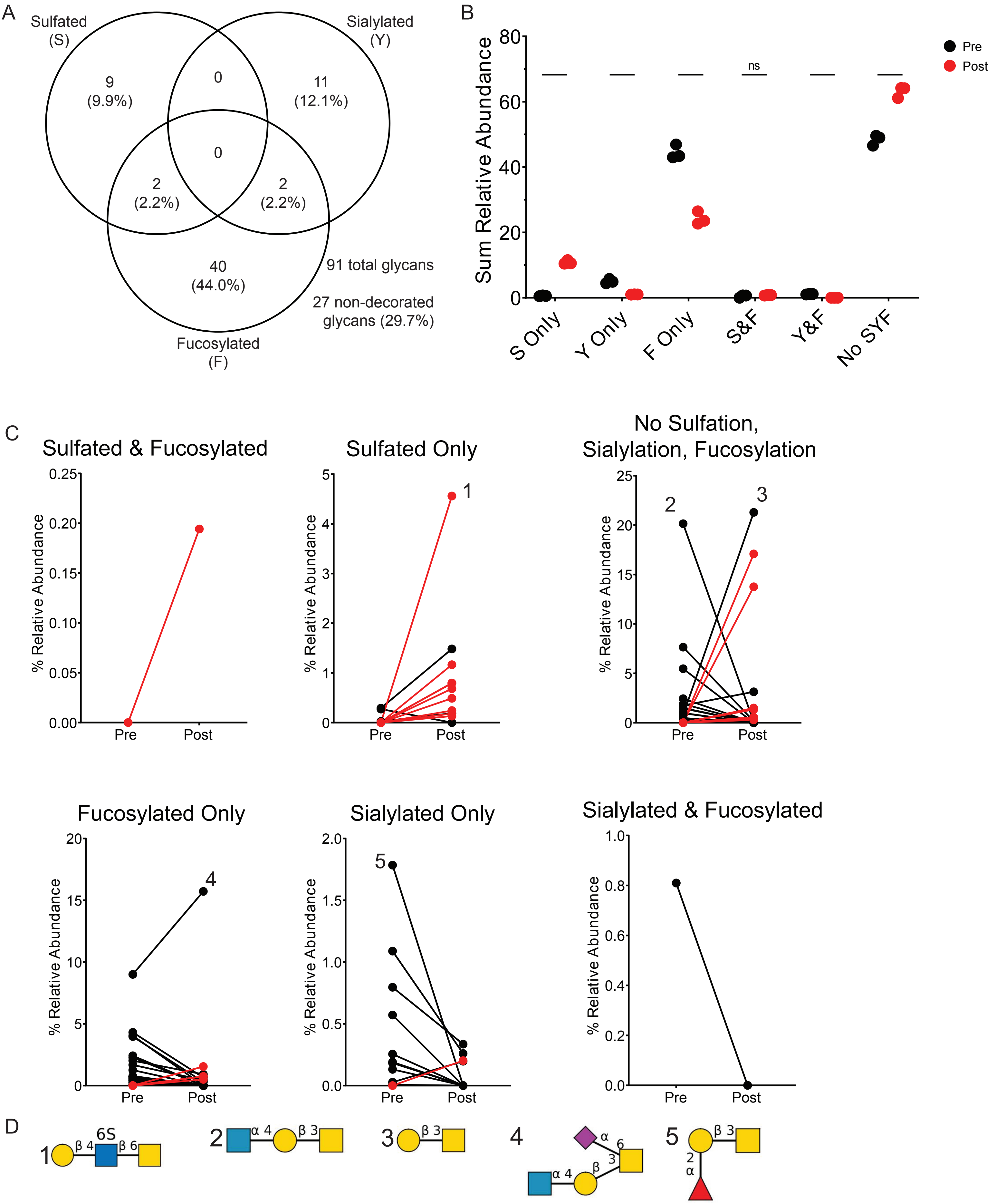

A
Sulfated (S)
Sialylated (Y)
Fucosylated (F)
9 (9.9%)
0
11 (12.1%)
0
2 (2.2%)
2 (2.2%)
40 (44.0%)
91 total glycans
27 non-decorated glycans (29.7%)
B
Sum Relative Abundance
Pre
Post
ns
S Only
Y Only
F Only
S&F
Y&F
No SYF
C
Sulfated & Fucosylated
Sulfated Only
No Sulfation, Sialylation, Fucosylation
Fucosylated Only
Sialylated Only
Sialylated & Fucosylated
% Relative Abundance
Pre
Post
D

Supplemental Figure S10. Fucosylated and sialylated glycans are degraded after growth of *R. torques* on PGM, but sulfated glycans accumulate. A, Number and percentage of total glycans detected in PGM pre- and/or post-growth of *R. torques* displaying indicated structural features by LC-MS/MS. **B**, Sum relative abundance of glycans and peeling reaction products pre- or post-*R. torques* growth on PGM with respective structural features. Statistical analysis was performed with paired, two-tailed t tests between pre- and post-growth samples for each structural feature category. S=sulfated, Y=sialylated, F=fucosylated. **$p<0.01$, ***$p<0.001$. **C**, relative abundance of glycans detected that were retained on the mucin polypeptide backbone and were significantly different in pre- or post-growth samples of *R. torques* on PGM. Red lines indicate glycans that were not detected in any of the pre-growth samples but were detected in the post-growth samples. Each point represents the average relative abundance of three samples. **D**, Putative structures of select detected glycans. Numbers refer to corresponding plot in C. Structures generated with DrawGlycan[69].

Figure S11

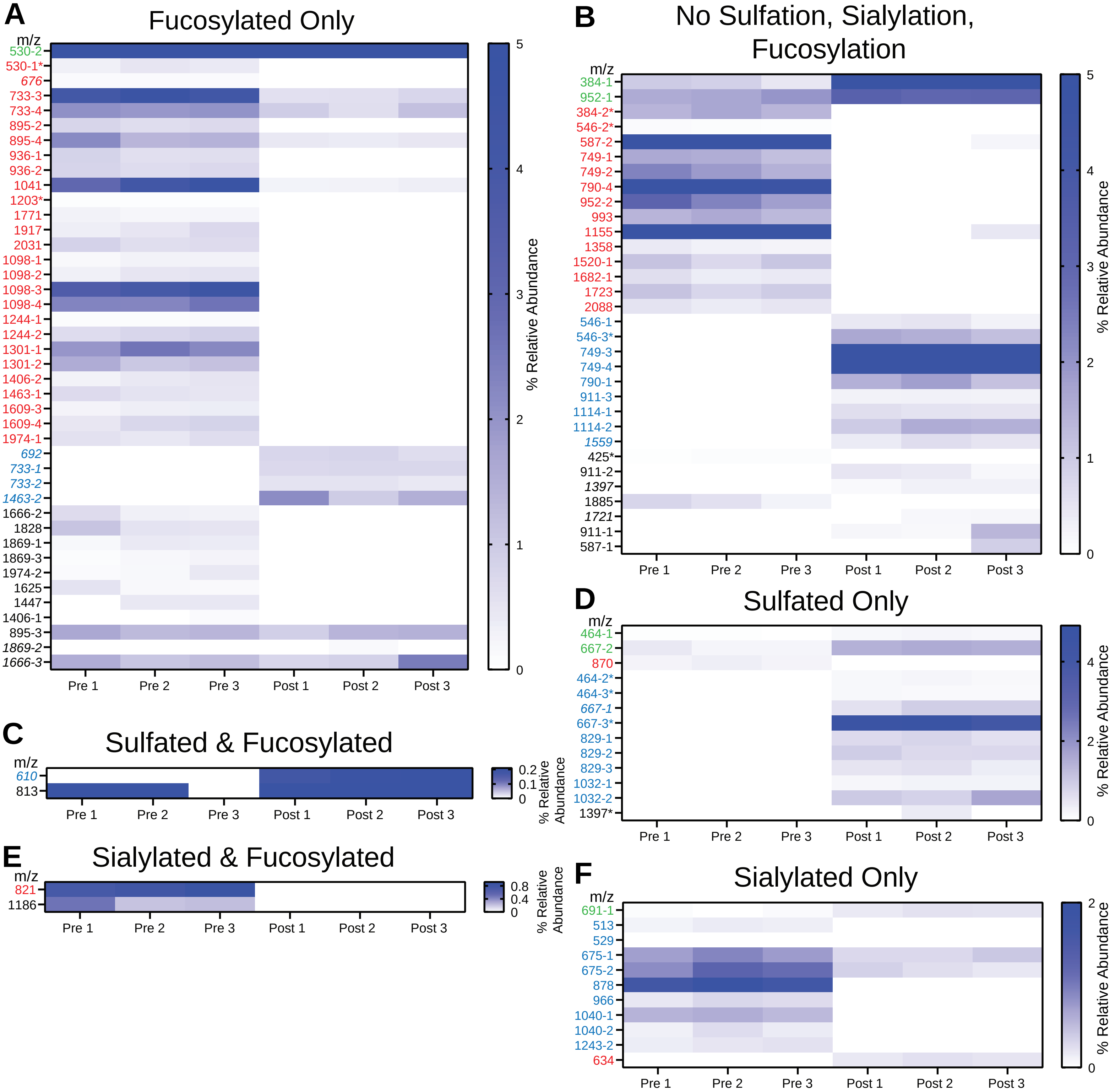

A
Fucosylated Only
m/z
530-2
530-1*
676
733-3
733-4
895-2
895-4
936-1
936-2
1041
1203*
1771
1917
2031
1098-1
1098-2
1098-3
1098-4
1244-1
1244-2
1301-1
1301-2
1406-2
1463-1
1609-3
1609-4
1974-1
692
733-1
733-2
1463-2
1666-2
1828
1869-1
1869-3
1974-2
1625
1447
1406-1
895-3
1869-2
1666-3
Pre 1
Pre 2
Pre 3
Post 1
Post 2
Post 3
% Relative Abundance
B
No Sulfation, Sialylation, Fucosylation
m/z
384-1
952-1
384-2*
546-2*
587-2
749-1
749-2
790-4
952-2
993
1155
1358
1520-1
1682-1
1723
2088
546-1
546-3*
749-3
749-4
790-1
911-3
1114-1
1114-2
1559
425*
911-2
1397
1885
1721
911-1
587-1
% Relative Abundance
C
Sulfated & Fucosylated
m/z
610
813
% Relative Abundance
D
Sulfated Only
m/z
464-1
667-2
870
464-2*
464-3*
667-1
667-3*
829-1
829-2
829-3
1032-1
1032-2
1397*
% Relative Abundance
E
Sialylated & Fucosylated
m/z
821
1186
% Relative Abundance
F
Sialylated Only
m/z
691-1
513
529
675-1
675-2
878
966
1040-1
1040-2
1243-2
634
% Relative Abundance

Supplemental Figure S11. LC-MS/MS glycans identified before and after growth of *R. torques* on PGM. A-F, Glycans detected were grouped based on structural features: fucosylated only (**A**), no sulfation, sialylation, or fucosylation (**B**), sulfated and fucosylated (**C**), sulfated only (**D**), sialylated and fucosylated (**E**), and sialylated only (**F**). Heat maps indicate percent relative abundance of each glycan pre- or post-growth of *R. torques.* Molecular weights in green represent glycans detected in at least one pre-growth sample that significantly increase in abundance post-growth; glycans in red decreased in abundance post-growth compared to pre-growth, glycans in blue were undetected pre-growth but were detected post-growth; and glycans in black were not significantly different in abundance pre- or post-growth. Statistics were analyzed using paired, two-tailed t-tests between the three pre- and three post-growth samples for each glycan. MW = molecular weight. * = peeling reaction product, italicized m/z values = *N*-glycan.

Figure S12

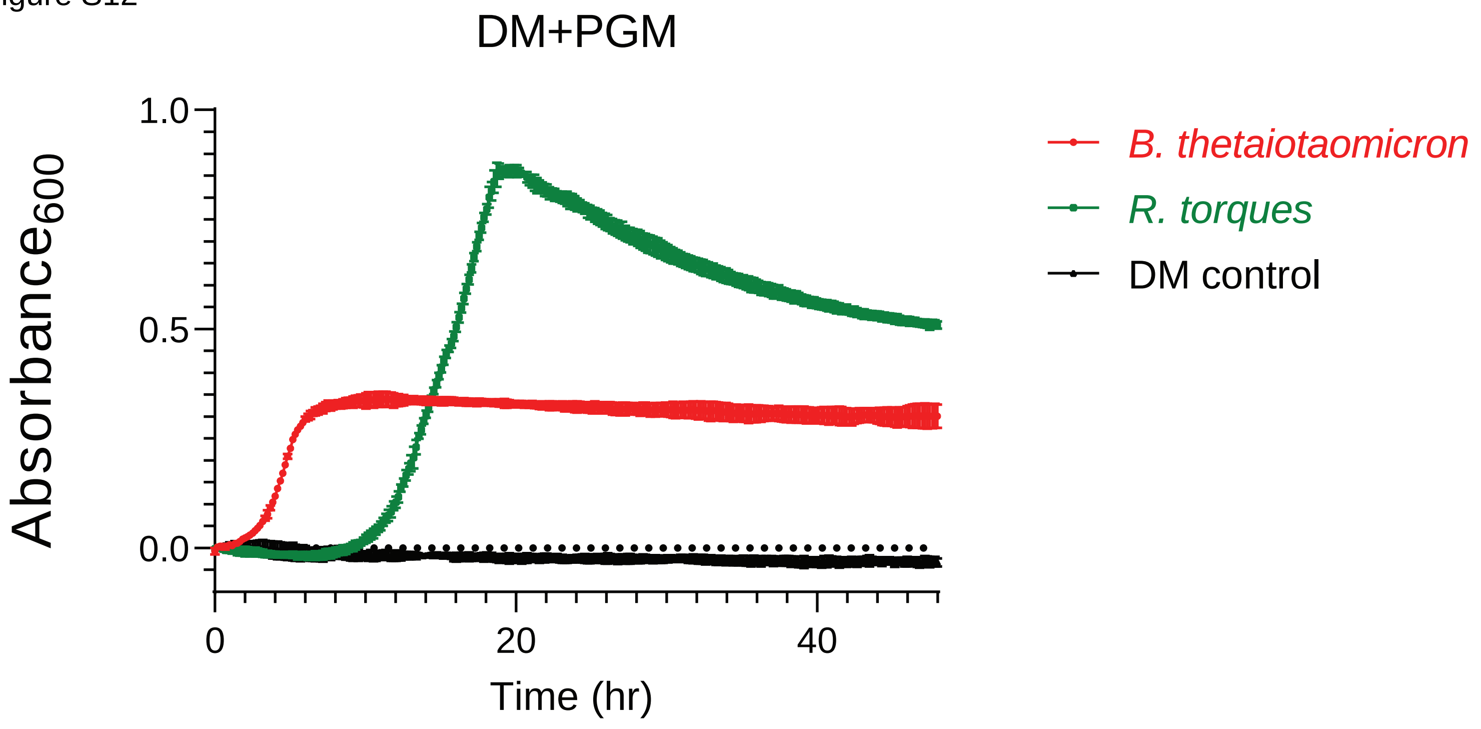
DM+PGM
1.0
0.5
0.0
Absorbance600
0
20
40
Time (hr)
B. thetaiotaomicron
R. torques
DM control

Supplemental Figure S12. Growth of *B. thetaiotaomicron* and *R. torques* on PGM in partially defined medium (DM). *B. thetaiotaomicron* tdk$^{-/-}$ and *R. torques* were grown anaerobically at 37ºC in DM with PGM (10 mg/ml final concentration) as the major carbohydrate source and growth was monitored by Absorbance$_{600}$. DM control contained DM+PGM only. Each point represents n=3 and error bars represent SD.

Figure S13

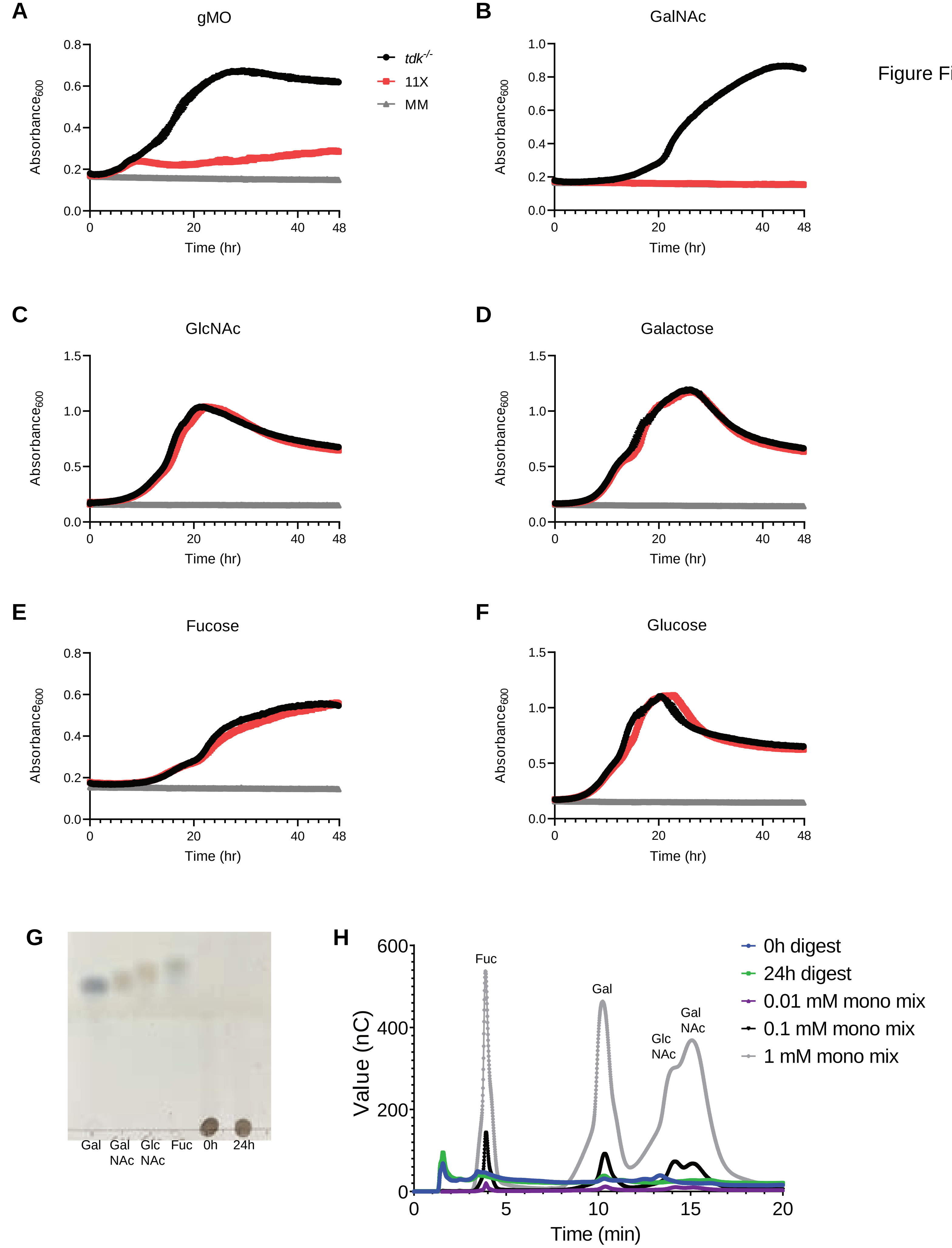
A
gMO
Absorbance600
Time (hr)
tdk-/-
11X
MM
B
GalNAc
Figure Fi
C
GlcNAc
D
Galactose
E
Fucose
F
Glucose
G
Gal
Gal NAc
Glc NAc
Fuc
0h
24h
H
Value (nC)
Time (min)
Fuc
Gal
Glc NAc
Gal NAc
0h digest
24h digest
0.01 mM mono mix
0.1 mM mono mix
1 mM mono mix

Supplemental Figure S13. Growth of *B. thetaiotaomicron* tdk$^{-/-}$ and *B. thetaiotaomicron* 11X mutant on glucose, gMO, and mucin monosaccharides and dialysis validation of *R. torques* PGM digest. A-F, Both strains of *B. thetaiotaomicron* were grown in Bacteroides minimal medium with gMO (**A**), *N*-acetylgalactosamine (**B**, GalNAc), *N*-acetylglucosamine (**C**, GlcNAc), galactose (**D**), fucose (**E**), or glucose (**F**). MM = Bacteroides minimal medium negative control. gMOs were at a final concentration of 10 mg/ml, and all others were 5 mg/ml. The first collected A_{600} value (0 h) was omitted from all conditions due to high baseline noise. Each point represents n=3 and error bars represent SD. **G**, thin layer chromatogram of *R. torques*-digested PGM substrates (0h control and 24h digest) to assess efficiency of dialysis in removing monosaccharides from these substrates. Standards at 10 mM were spotted once (3 µl), PGM substrates at 20 mg/ml were spotted twice (3 µl each). Gal=galactose, GalNAc=*N*-acetylgalactosamine, GlcNAc=*N*-acetylglucosamine, Fuc=fucose. **H**, HPAEC-PAD analysis of 0h and 24h PGM digests for presence of monosaccharides after dialysis. Mono mix=fucose (fuc), galactose (gal), *N*-acetylgalactosamine (GalNAc), and *N*-acetylglucosamine (GlcNAc). Concentrations for mono mixes reflect concentration of each monosaccharide in the mix.
